# Influence of Micro‐Scale Surface Texturing on Light Distribution From Luminescent Downshifting Layers for Next‐Generation Greenhouses

**DOI:** 10.1002/gch2.70163

**Published:** 2026-09-25

**Authors:** Juvet N. Fru, Boris Breiner, Monica S. Saavedra, Stephanie Sheppard, Bryce S. Richards

**Affiliations:** ^1^ Institute of Microstructure Technology Karlsruhe Institute of Technology (KIT) Eggenstein‐Leopoldshafen Germany; ^2^ Lambda Energy Ltd. Crop Technology Southern Innovation Hub, College Road Cranfield UK; ^3^ Light Technology Institute Karlsruhe Institute of Technology (KIT) Karlsruhe Germany

**Keywords:** luminescent downshifting layers, Monte Carlo ray‐tracing, next‐generation greenhouses, PAR spectrum

## Abstract

Luminescent downshifting (LDS) layers in greenhouses modify the photosynthetically active radiation (PAR) spectrum to enhance plant growth, with surface texturing enabling a critical role in improving downward light extraction. The 3 mm‐thick LDS layers incorporate two different dyes (Lumogen F Red 305 and Eu‐coordination complex) and six micro‐structures (cones, square pyramids, cylinders, half‐cylinders, domes, and prisms) are systematically optimised to maximise downward‐extracted (*DE*) efficiency of both converted red luminescence and unconverted (not absorbed by luminophores) sunlight. Monte Carlo ray‐tracing simulations model small‐area (25 cm^2^) and infinite‐area LDS layers and their integration into a 10 × 4 × 2 m greenhouse. The highest *DE* efficiency (65%) occurs in the infinite LDS layers containing bottom‐indented cylinders with an aspect ratio (*AR*) of 0.9. These results emphasise the importance of moving beyond small‐area LDS layer performance to determine the amount of red light (converted luminescence as well as unconverted sunlight) that reaches the floor of a full‐scale greenhouse, defined here as the red enhancement fraction (*REF*). The *REF* is maximised for a greenhouse containing Lumogen F Red 305 in Karlsruhe in winter (70%) using top‐indented domes and bottom‐indented cylinders (*AR* = 0.2), while smaller gains are made in summer (22%) and in spring/autumn (35%).

## Graphical abstract

This work investigates two different luminophores that convert ultraviolet to red light along with a wide range of microstructures to maximise outcoupling of the luminescence as well as the incoming solar radiation onto the greenhouse floor. Starting with small‐area (25 cm^2^) luminescent down‐shifting (LDS) layers, the best designs are then evaluated for a full‐size greenhouse (40 m^2^ floor area).

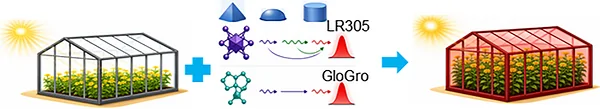

## 1 Introduction

**FIGURE 1:**
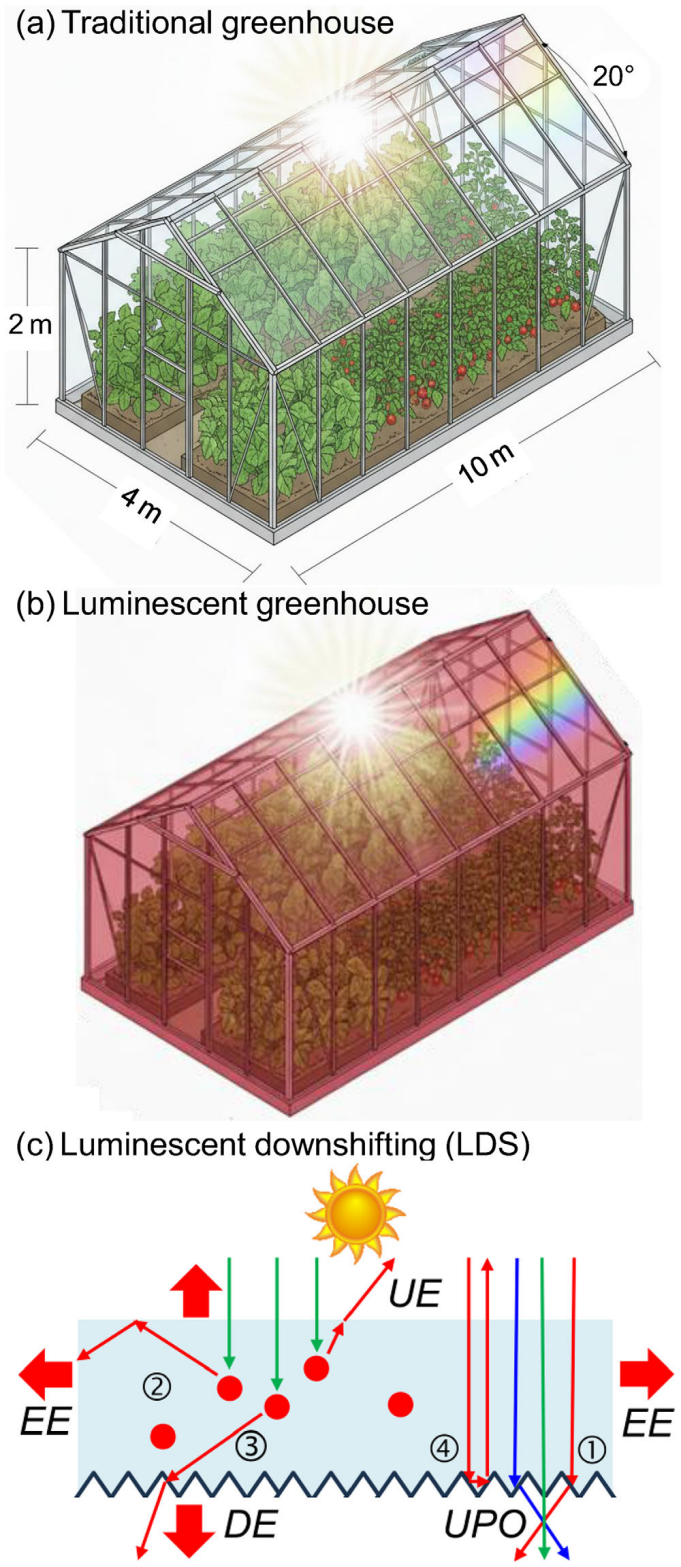
Schematic diagram of the simulated: (a) conventional dye‐free planar greenhouse with 10 × 4 × 2 m dimension and roof inclination of 20°: (b) Luminescent greenhouse of the same dimensions, featuring dye‐doped sheets exhibiting a micro‐texture to enhance light out‐coupling to the greenhouse floor; (c) A micro‐textured LDS slab containing red‐emitting dye molecules (,) illustrating four different ray pathways (①—④, described in the main text) and the desired down extraction (*DE*) direction—as opposed to edge extraction (*EE*) and upward extraction (*UE*), which are both losses. The unconverted photon outcoupling (*UPO*) efficiency, showing the fates of various incoming wavelengths of sunlight that are not absorbed by the dye are also illustrated.

The global population has increased from 3.0 billion in 1960 to 8.3 billion in 2025 and is projected to reach 9.7 billion by 2050, potentially peaking near 10.3 billion around 2084 [1, 2]. This rapid growth, followed by gradual deceleration, places unprecedented pressure on food security and energy resources. With this in mind, the Food and Agriculture Organization (FAO) of the United Nations highlights that improving agricultural productivity sustainably is needed to provide the extra food required as populations rise around the world [3]. Traditional greenhouses (Figure 1a) can help ensure food security via creating a controlled environment that enables crop production, even in challenging climates. To achieve this sustainably requires operating within the food‐energy‐water nexus while focusing on four key factors: light, temperature, carbon dioxide levels, and water availability. Next‐generation greenhouses are predicted to offer advances in broad‐range spectral management of sunlight to optimize crop growth in a net‐zero energy manner [4]. For example, i) the photosynthetically active radiation (PAR) spectrum (400 – 700 nm) is prioritized for plant growth [5]; ii) under‐utilized wavelengths in the ultraviolet (UV, 300 – 400 nm) and green can be converted into additional blue and red photons to boost the PAR spectrum [6, 7]; iii) harvesting near‐infrared (700 – 1200 nm) for electricity generation via silicon‐based photovoltaic modules [8]; and iv) managing infrared bands for dynamic thermal control, either heating or via passive daytime radiative cooling processes [9].

Luminescent downshifting (LDS) layers greenhouses (Figure 1b) offer a promising solution by converting the less‐utilized green and UV sunlight into additional PAR photons in the blue (400 – 500 nm) and red (600 – 700 nm) wavelengths [10, 11]. This photon management process enhances light‐use efficiency in plants (LUE) [6, 12], resulting to improved crop quality [6], growth [11, 13], and yield [12, 13], and is thus a promising sustainable (zero energy consumption) method for enhancing crop production and quality. For instance, Bergren et al. investigated CuInS_2_/ZnS quantum dot films for greenhouse applications to optimize the solar spectrum and improve plant growth efficiency without additional electricity consumption [6]. The films convert UV and blue photons into ∼600 nm emission, reducing the daily light integral (DLI)—a measure of the total amount of PAR light a plant receives in a 24 h period—by 14%. However, despite the reduced DLI, the results indicate a 10% faster vegetative growth rate, a 36% reduction in fruit waste, and a 5.7% increase in saleable yield per unit area. Additionally, plants exhibit a 23% increase in LUE normalized to total DLI, indicating improved production efficiency [6].

A planar LDS system is formed via embedding a luminescent species into a transparent planar slab, typically with a refractive index of n ≈ 1.5. This configuration commonly referred to as luminescent solar concentrator [14, 15], whereby total internal reflection (TIR) waveguides most of the emitted light to the edges, while only a small fraction of the luminescence is directed downward via the escape cone losses [15, 16, 17], theoretically 13% for a matrix with *n* ≈1.5. Practically, these escape cone losses can be much higher and depend on the overlap between the absorption and emission spectra of the dye (Stokes shift) as well as the dye concentration [18] In addition, in a symmetrical system, the same amount of luminescence will depart via the front‐side escape cone, a clear loss for the intended greenhouse application.

In next‐generation greenhouses that use luminescent solar concentrators, photovoltaic cells attached at the edges capture a higher proportion of the converted light to generate electricity, while only a limited fraction of the converted light transmits downward for plant growth [19]. However, textured LDS layers operate differently. These layers aim to outcouple most converted photons downward rather than guide them to the edges, thereby enhancing downward light extraction.

To enhance the downward extraction (*DE*) efficiency of the converted light, the system must disrupt TIR. Disrupting TIR increases both *DE* and upward extraction (*UE*) efficiencies, but reduces edge extraction (*EE*) efficiency. For greenhouse applications, *EE* should be minimized and *DE* should ideally be much greater than *UE*. Surface texturing of the top and/or bottom surfaces [20, 21, 22] or the incorporating scattering particles within the bulk matrix [23] can achieve this effect. This study focuses on improving the *DE* of converted photons via surface texturing. Figure 1b illustrates an LDS layer with micro‐textures on the rear side, illustrating how this affects: ① the direct transmission of an unconverted ray (defined as being a ray that is not absorbed by the dye); ② TIR of a converted ray; ③ the disruption of TIR of converted ray by surface textures; and ④ losses introduced via some surface textures that exhibit retroreflection. The *DE*, *UE*, and *EE* efficiencies represent the percentage of emitted photons extracted in the downward, upward, and edge directions, respectively, with *DE* being the only desired outcome.

Previous studies have also explored *DE* efficiency enhancement in LDS‐based greenhouse systems through surface texturing. For example, Shen et al. used Monte Carlo ray tracing to model a polymethyl‐methacrylate (*n* = 1.5) LDS layer, 0.075 mm thick and 100 mm^2^ in area, doped with Lumogen F Red 305 (LR305) dye that exhibits a primary absorption peak at 576 nm and an emission peak at 612 nm [20]. The optimum design featured a square lattice of micro‐domes on the top surface, with a diameter 400 µm and a height 65 µm—i.e. an aspect ratio (*AR*) of 0.33. This configuration achieved a *DE* efficiency of 65%, compared with 13% for planar films, and improved yield [20, 21]. In addition, the design increased lettuce fresh weight by 55%–66% per mole of light per square meter.

However, this design has some limitations. First, it attenuated blue light, which is important for photosynthesis [20]. The film absorbed nearly 45% of blue light (400–500 nm range) due to the non‐negligible absorption of LR305 fluorophores in this region. This reduction altered lettuce morphology. Second, the large feature size (400 µm) on top surface increases the risk of soiling due to dust accumulation [24]. Although micro‐textured top surfaces can exhibit superhydrophobic behavior and provide functionalities such as self‐cleaning and antireflection, dust particles smaller than the texture features can become trapped within surface cavities, making them difficult to remove [24]. Third, micro‐structures on the top surface are susceptible to severe abrasion under harsh environmental conditions [25, 26], which reduce will optical transmission and suppress *DE* efficiency.

To counteract the potential effects of severe abrasion and soiling when large size micro‐structures are on the top surface, Xu et al. proposed a structurally‐protected enhancement approach that completely avoids surface top surface structures of any size by integrating bottom‐indented, uniform micro‐cone arrays into horticultural LDS layers [22]. For a 3 mm‐thick sample with an area of 6400 mm^2^ and *n* = 1.5 doped with LR305 dye, Monte Carlo ray‐tracing simulations indicated that a *DE* efficiency of 43% could be achieved with micro‐cones with an *AR* of 3, a fixed radius of 50 µm, and a fluorophore concentration of 2 × 10^−6^ M. However, textures on the bottom surface may cause retroreflection (see ④ in Figure 1c), an optical phenomenon in which light reflects directly back toward its source [27]. This effect would significantly reduce the *UPO* efficiency and results in extremely low average irradiance. The reduction in available light limits photosynthetic activity and constrains crop growth, ultimately lowering overall productivity [28].

The research community continues to address the parasitic attenuation of blue light intensity associated with the use of LR305. One approach involves exploring luminophores with large Stokes shifts that absorb in the UV region and emit in the red region [11]. For example, Müller et al. investigated Eu^3+^‐containing polyoxotitanate (Eu‐POT) LDS materials for greenhouse applications to enhance crop productivity and support sustainable agriculture [11]. The system converts UV radiation into red light with a high PLQY (68%) through a systematically optimized molecular design. The researchers incorporated the LDS compound into a water‐based acrylic varnish that can be spray‐coated onto greenhouse surfaces, enabling practical deployment. Although the study does not fully isolate the contribution of the downshifting effect, treated plants showed measurable improvements, including a 9% increase in basil leaf dry weight and a 10% increase in individual leaf dry mass. These results demonstrate the potential of LDS coatings to improve plant growth efficiency. Despite these promising results, the authors found out that Eu‐POT exhibited limitations in long‐term stability and high cost, which may restrict its large‐scale commercial implementation. Consequently, the authors developed a proprietary Eu‐based coordination complex (GloGro) with improved stability and substantially lower production costs. This next‐generation luminophore is one of the materials investigated in the present study.

Another approach involves developing UV‐to‐blue phosphors [29, 30, 31, 32] that can complement the LR305 dye within a single design. In such systems, UV‐to‐blue phosphors can compensate for blue light that is intrinsically attenuated by LR305 absorption. Yousif et al. investigated the enhancement of PLQY in Bi^3+^‐ doped CaYGaO_4_ phosphors through the optimization of lithium‐based flux additives [30]. By comparing Li_2_O and Li_2_CO_3_ fluxes, the study identified distinct structural and optical effects. Samples treated with Li_2_CO_3_ showed superior crystallinity and reduced oxygen‐related defects, resulting in a PLQY of 70%, which outperformed flux‐free and Li_2_O‐treated samples.

More recently, Xu et al. (2025) conducted a comprehensive optical loss‐analysis and light‐extraction optimization study of horticulture LDS layers, analyzing their performance, loss mechanisms, and strategies to enhance light extraction efficiency [33]. A theoretical framework was established through derived equations and subsequently validated using Monte Carlo simulations and experimental data. The study highlights that managing and extracting unconverted direct red light (see ① in Figure 1b) is equally critical for maximizing overall red photon output in horticultural applications. It also introduces total red extraction—comprised of the unconverted red wavelengths coming from sunlight directly plus the shorter‐wavelength light that was absorbed and converted to red luminescence—as a key performance metric that represents the overall efficiency of LDS in delivering usable red light to plants that has to be maximized.

Despite these advances, several knowledge gaps remain. Shen et al. provided a detailed quantitative analysis of transmittance losses due to increased Fresnel reflection but focused primarily on top‐protruding macro‐domes. The authors did not explore other geometries, surface positions (top vs. bottom), aspect ratios, or orientations (indented vs. protruding), all of which may influence transmittance through scattering, trapping, and/or retroreflection of unconverted photons, even at low incident angles. Furthermore, most studies focus on optimizing the performance of LDS layers in isolation and do not evaluate how these structures perform once used for the construction of a full‐scale LDS greenhouse under realistic solar irradiation conditions. Such conditions may introduce additional losses, including increased Fresnel losses at non‐normal incidence, scattering, trapping and/or retroreflection of unconverted photons that prevents light from reaching the greenhouse floor. Therefore, it is critical to comprehensively quantify red‐light enhancement, average irradiance and irradiance homogeneity (Figure S1) at greenhouse floor level across different times of day and seasons when optimizing LDS layers.

More generally, molecular luminophores such as LR305 and GloGro are particularly appropriate for transparent greenhouse LDS layers because their molecular‐scale dimensions are far smaller than visible wavelengths. When molecularly dispersed, they cause negligible particle‐driven Mie scattering and thus help maintain the optical transparency of the LDS matrix. LR305 is an organic molecular dye noted for broad absorption, a high photoluminescence quantum yield (PLQY), and good photostability [34], while GloGro is a molecular europium‐based coordination complex characterized by selective ultraviolet absorption, a large Stokes shift, and narrow red emission. Other luminophore groups include quantum dots (QDs) alongside inorganic phosphors. Quantum dots can show high PLQYs as well as composition‐dependent, tunable absorption and emission spectra; however, their use may be constrained by relatively small Stokes shifts, limited long‐term photostability, and, depending on the composition, the presence of toxic elements that may be inappropriate for agricultural applications [6, 35]. Inorganic phosphors can deliver high PLQYs, excellent photochemical stability, and emission wavelengths that cover the visible spectrum, including UV‐to‐blue and orange/red conversion [29, 30, 31, 32]. Nevertheless, their commonly micrometer‐scale particle sizes can lead to extra optical scattering within the LDS matrix, which may have a negative effect on the amount of PAR reaching the greenhouse floor. Taken together, these complementary attributes indicate that selecting luminophores for greenhouse LDS applications should involve not only spectral conversion efficiency but also the Stokes shift, photostability, toxicity, particle size, and the luminophore's effect on light propagation within the LDS layer.

**FIGURE 2:**
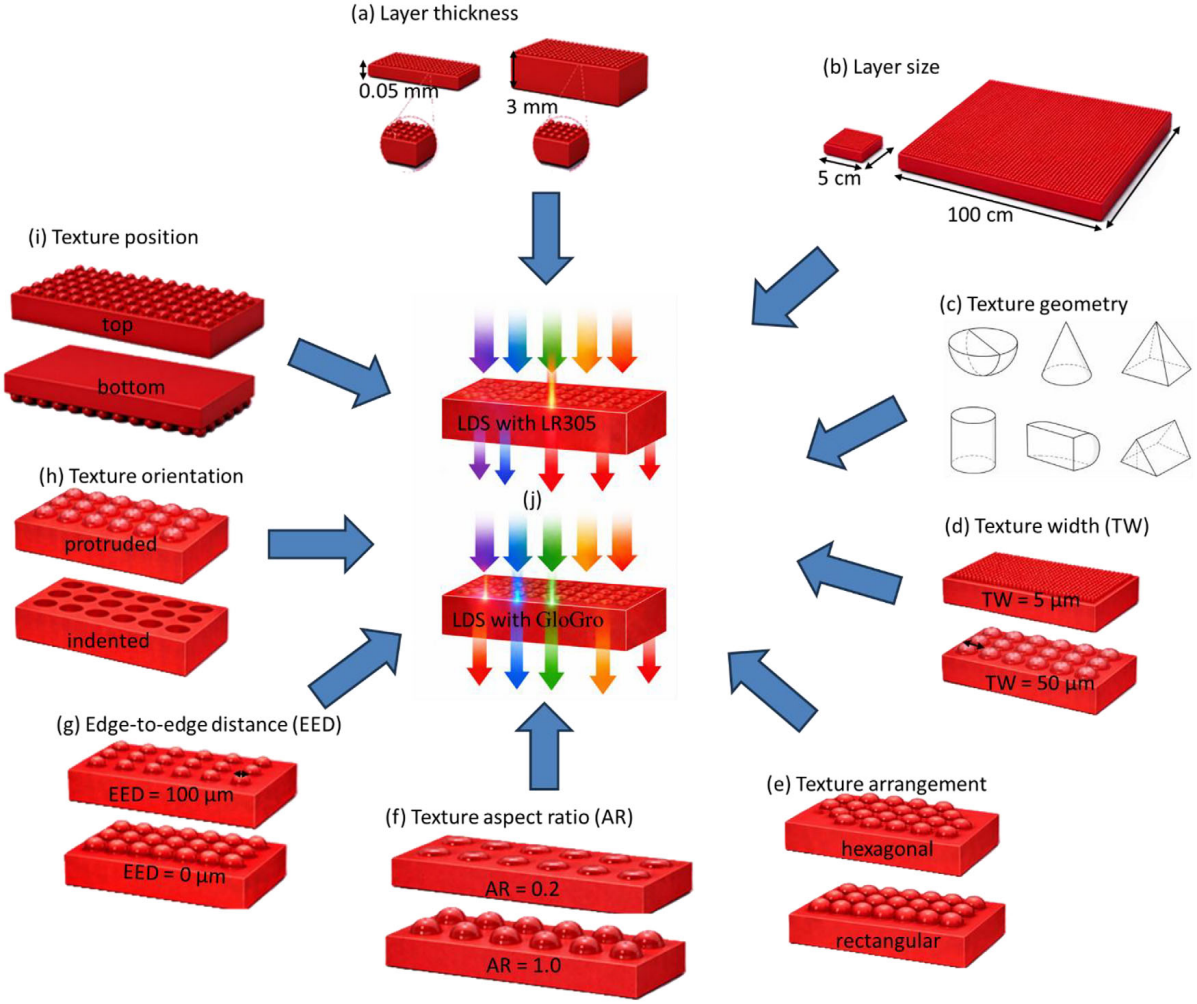
Schematic illustration of the key design parameters investigated for micro‐textured luminescent downshifting layers (refractive index, *n* = 1.5). These include (a) layer size (5–100 cm), (b) layer thickness (0.05–3 mm), (c) micro‐texture geometry (domes, cones, square pyramids, cylinders, half‐cylinders, and prisms), (d) feature base width (0–100 µm), (e) packing arrangement (rectangular or hexagonal), (f) aspect ratio (*AR* = 0–1.8), (g) edge‐to‐edge spacing between features (0–100 µm), (h) feature orientation (indented or protruded), (i) feature position (top or bottom surface), and (j) dye type (LR305 or GloGro).

This work explicitly accounts for the net effects of scattering, trapping, and/or retroreflection of unconverted photons by six micro‐textures (e.g. domes, cones, pyramids, cylinders, half‐cylinders, and prisms) through unconverted photon outcoupling (*UPO*) efficiency. These geometries were selected to represent distinct surface characteristics, including curvature, faceting, the presence or absence of apices, edges or ridges, and different degrees of symmetry and directionality (isotropic versus anisotropic), which can influence light extraction and redistribution. In addition, the six geometries are compatible with scalable replication techniques such as hot embossing [36], which offers a promising route for producing high‐fidelity micro‐structured surfaces over large areas [37]. The work systematically evaluates how many parameters—summarized in Figure 2a–j—influence the *DE* efficiency of converted red photons and the *UPO* efficiency in LDS layers with n = 1.5. Importantly, the present study also includes feature widths in the 5–50 µm range, which, to the best of the authors’ knowledge, have not previously been investigated for textured LDS layers. The smaller features can provide photon‐extraction performance comparable to that of larger features with the same aspect ratio (*AR*) while reducing the surface‐relief height. The lower surface relief may reduce the exposure of protruding micro‐structures to mechanical contact, thereby reducing their susceptibility to abrasion and surface damage, while the smaller lateral dimensions allow a greater number of features to be accommodated within a given surface area, providing greater flexibility in controlling texture density, spacing, and arrangement.

The study also evaluates red‐light enhancement in an LDS greenhouse through the red enhancement fraction (*REF*) and quantifies spatial, and angular light distributions at the floor. It provides benchmarks for surface‐textured LDS greenhouses evaluated from sunrise to sunset at a single geographic location (Karlsruhe, Germany) on three representative days of the year (equinox and solstices). Collectively, these analyses establish an integrated framework linking LDS layer micro‐texture design, downward outcoupling efficiency of converted photons, micro‐structure‐induced optical trapping and retroreflection of unconverted photons, and real‐world light management. This framework provides a pathway to maximize photon extraction and distribution for photosynthesis while minimizing light losses and structural vulnerabilities in LDS greenhouse applications.

The novelty of the present work, compared with existing knowledge, is as follows. First, six geometrically distinct micro‐structures (domes, cones, pyramids, cylinders, half‐cylinders, and prisms) are systematically compared under identical LDS optical and geometrical conditions, enabling the influence of geometry on optical performance to be isolated. Notably, to the best of the authors’ knowledge, cylindrical and half‐cylindrical textures have not previously been systematically investigated as surface extraction structures specifically for LDS layers. Second, the analysis considers not only the extraction of converted luminescent photons but also the micro‐structure‐induced redirection, trapping, and retroreflection of useful unconverted photons, thereby revealing that enhanced luminescent extraction does not necessarily maximize the total useful light delivered to the greenhouse. Third, the study extends the analysis from conventional cm^2^‐scale LDS layers to a full‐scale, m^2^‐scale greenhouse, enabling the optimized micro‐structures to be evaluated over the meter‐scale propagation distance from the greenhouse roof to the crop level under realistic solar trajectories. This multiscale analysis establishes how micro‐structure‐dependent photon extraction and redistribution at the LDS‐layer level translate into angular and spatial light distributions, PAR availability, and overall light delivery at the greenhouse floor, thereby providing design guidance that cannot be obtained from layer‐level extraction efficiency alone.

## 2 Methods

The models of the small‐scale textured LDS layers and full‐scale model of the LDS greenhouse used for LightTools simulations are illustrated in Figure 1. The textured LDS layers were used for experimenting with a wide range of parameters, as follows:

- *Size:* LDS layer dimensions ranging from 5 to 100 cm;
- *Thickness:* LDS layer thickness ranging from 0.05 to 3 mm;
- *Geometry*: a range of micro‐texture geometries, including domes (Do), cones (Co), square pyramids (Py), cylinders (Cyl), half‐cylinders (H‐Cyl), and prisms (Pri);
- *Width*: feature base width ranging from 0.0 to 100 µm, where a width of 0.0 µm represents an untextured layer with no surface structures;
- *Packing arrangement*: rectangular or hexagonal;
- *AR*: ranging from 0.0 – 1.8 (i.e. also capturing the feature height);
- *Edge‐to‐edge distance (EED)*: of features: ranging from 0.0 to 100 µm, where 0.0 µm corresponds to the condition in which the edges of adjacent features just touch;
- *Orientation*: indented (I) or protruding (P);
- *Position* : textures located on either the top or bottom surface of the LDS layer;
- *Dye type*: LR305 dye or GloGro.

Further details are provided below.

### 2.1 LDS Materials

The host material for the LDS layers was chosen to have a *n* = 1.5, consistent with glass, a commonly used greenhouse material. A nondispersive refractive index was assumed for simplicity. Except for the thickness study, all sheets have a thickness of 3 mm to reflect standard commercial greenhouse glazing [38]. Two distinct luminescent species were chosen as the optically active components of the LDS layers. First, the perylene‐based Lumogen F Red 305 dye (LR305; BASF, Germany) was selected because it is a commercially available organic dye with relatively broad absorption that extends from the blue into the orange spectral region, strong red emission, a near‐unity PLQY, and excellent photostability [34]. Second, the europium‐based coordination complex GloGro was selected because it selectively absorbs ultraviolet light, shows a large Stokes shift, yields a narrow emission band centered at approximately 612 nm, has a PLQY of 65%, and provides improved stability along with lower production costs. GloGro was developed as a proprietary Eu‐based coordination complex with enhanced stability and substantially lower production costs than the previously reported Eu‐POT system [11]. These two luminophores were selected to deliver contrasting spectral‐conversion characteristics, so that the effect of surface micro‐structuring on light extraction and distribution could be assessed across different luminophore systems without introducing additional material‐specific variables.

**FIGURE 3:**
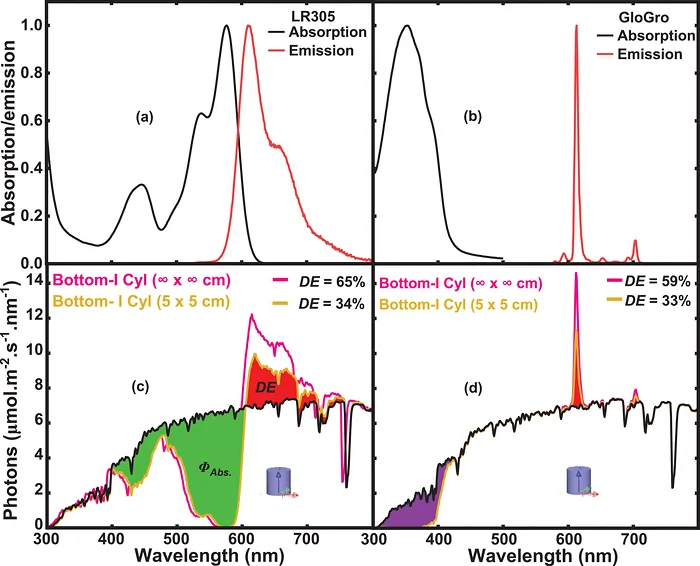
(a) Normalised absorption and emission spectra of Lumogen Red 305 (LR305) dye (data taken from [34]); (b) Normalised absorption and emission spectra of Eu‐coordination complex, GloGro, luminophore (data taken from [11]); Spectral irradiance of 5 × 5 × 0.3 cm and ∞ × ∞ × 0.3 cm textured LDS layers with bottom‐indented cylinders at an aspect ratio of 0.9 (bottom‐I Cyl; *AR* = 0.9) layers (c) using LR305 dye, showing absorbed photons (green) and downward extracted photons (pink). The spectra also illustrate the enhancement relative to the planar layer (Figure S5a); and (d) using GloGro luminophore, showing absorbed photons (purple) and downward extracted photons (pink).

The absorption and emission profiles of both luminescent materials are plotted in Figure 3a,b. The absorption spectrum of LR305 used in this work was obtained from Zang et al. [34] and was measured at a dilute concentration (0.0001 mM) to ensure negligible reabsorption and aggregation effects. The spectrum was normalized, as required by LightTools, and implemented to define the spectral absorption profile. The correlation between simulated and actual dye loadings is illustrated in Figure S2. Unless otherwise stated, a LR305 dye loading of 375 ppm was used in this work, while a loading of 750 ppm was used for the validation experiment. Similarly, the absorption profile of GloGro was obtained from Müller et al. [11], with a loading of 375 ppm used throughout the study.

### 2.2 LDS Layer and Sheet Dimensions

The simulated small‐scale LDS layers measured 5 × 5 × 0.3 cm and these were also expanded to infinite sheets (∞ × ∞ × 0.3 cm) by adding perfect mirror properties at the edges of smaller sheets. This was done to have a performance indication for the larger sheets (each exceeding 100 cm × 100 cm) required later for the full‐size greenhouse model. The dimensions of the latter were 10 m in length, 4 m in width, and 2 m in height, with a roof inclination angle of 20°, as illustrated in earlier in Figure 1a. The roof sheet dimensions were 2.50 × 2.13 m. Sheets along the width measured 2.0 × 1.1 m, and sheets along the length measured 2.0 × 1.1 m. The roof support formed an isosceles triangle with a base of 4.0 m and a base angle of 20°.

### 2.3 Luminescent Downshifting Simulations

Optical simulations were performed using the Monte Carlo ray‐tracing algorithm in LightTools 2026 (Keysight Technologies, Santa Rosa, CA, USA). All small‐scale LDS layer geometries were drawn and textured in LightTools, except during comparisons with geometries drawn in SolidWorks 2025 (Dassault Systèmes SolidWorks Corporation, Waltham, MA, USA). For the full‐scale greenhouse, the geometries were drawn in SolidWorks and textured in LightTools. The air mass 1.5 global (AM1.5G) terrestrial solar spectrum in LightTools served as the illumination source. The luminescence simulations used the normalized absorption spectrum, normalized emission spectrum, PLQY of the dyes as inputs, along with the photon mean free path (*MFP*) as inputs. The *MFP* (Equation S1) data was determined from the normalized absorbance and layer thickness. Receivers for the LDS layers were positioned at a fixed distance of 1 µm from the planar surface or from the apex of protruding micro‐structures. This configuration enabled the determination of irradiance maps (spatial intensity distributions), angular irradiance distributions, and spectral distributions. For the small‐scale LDS layers, a large number of rays (10 million) ensured statistical accuracy. The light source power was 2.5 W, corresponding to an irradiance of 1000 W m^−2^ over an area of 25 cm^2^ when the source was normal to the surface. The lower detector recorded 920 W m^−2^ for the dye‐free planar layer due to Fresnel reflection losses at the top and bottom surfaces of the LDS layer. Normal irradiation was used for the small‐scale LDS samples to mimic standard laboratory characterization conditions. The *DE* efficiency of converted photons was calculated from the spectral irradiance plot (Figure 3c,d), as defined in Equation S2. The *UPO* efficiency for the small‐scale LDS layers—defined as the percentage ratio of the average transmitted irradiance to the incident reference irradiance—was computed using Equation S3. For the greenhouse configuration, 50 million rays were used in full‐scale greenhouse simulations to improve statistical accuracy. A receiver was placed 1 µm above the floor, and this small separation minimized undetected ray losses. The *REF* of the full‐scale LDS greenhouses was calculated as defined in Equation S4. The uniformity (*U*) of the spatial irradiance distribution on the greenhouse floor was calculated using the method described by Matsumori et al. [39]. To enable reproducibility of these results, three LightTools simulation files are included as Supporting Information (Sections S3.1 – S3.3 provides the corresponding file names and descriptions). To validate the optical simulation results, one configuration from the small‐scale LDS system (Py, *AR* = 1.4) was realized experimentally. A detailed description of the methods employed and results obtained (Figures S3a,b and S4a,b) appears in Section S5.

## 3 Results and Discussion

### 3.1 Small‐Scale Textured Luminescent Downshifting Layer Simulations (Under Normal Irradiation)

#### 3.1.1 Downward Outcoupling of Converted Red Photons (*DE* Efficiency)

The downward outcoupling of converted red photons from the LDS layer is characterized by the *DE* efficiency. When edge reflectors are introduced to an infinite lateral (∞ × ∞ × 0.3 cm) bottom‐I Cyl LDS layer, the *DE* efficiency experiences the most significant rise from 34% to 65% for LR305 and from 33% to 59% for GloGro (Figure 3c,d), respectively. The edge reflectors enhance *DE* efficiency by promoting photon recycling and increasing the optical path length, thereby increasing the frequency of photon–structure interactions. The enhancement is greater for LR305 than for GloGro. This difference arises because LR305 absorbs in the green region of the spectrum, where photon flux is higher than in the UV region absorbed by GloGro. Consequently, LR305 generates a higher density of converted red photons relative to the number of micro‐structures, leading to more frequent photon–structure interactions and more effective TIR disruption. However, overall, it should be noted that the fraction of edge‐escaping photons remains high in the 25 cm^2^‐area LDS layers and the *DE* efficiency can be nearly doubled by simply upscaling (in this case to an infinite sheet).

**FIGURE 4:**
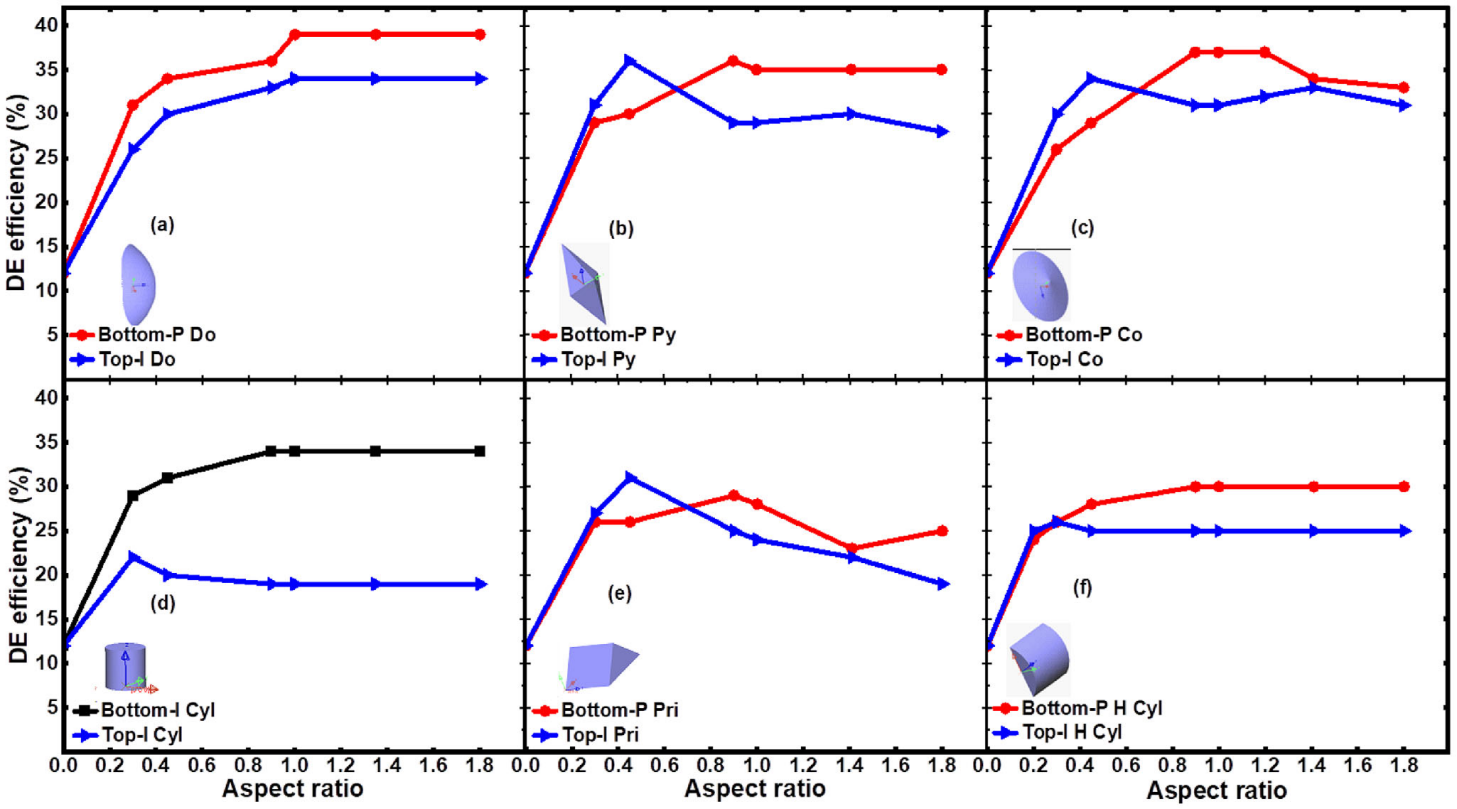
Downward extraction (*DE*) efficiency of converted photons as a function of aspect ratio (*AR*; horizontal axis), surface position, and orientation (curves) for different micro‐structure geometries: (a) dome (Do), (b) pyramid (Py), (c) cone (Co), (d) cylinder (Cyl), (e) prism (Pri), and (f) half‐cylinder (H‐Cyl). Bottom‐P and top‐I denote bottom‐protruding and top‐indented micro‐structures, respectively.

Figure 4a–f illustrates how the *DE* efficiency on the 25 cm^2^‐area LDS layers varies with *AR*, surface location, and orientation for six different micro‐structures—Do, Py, Co, Cyl, Pri, and H‐Cyl—with a constant width of 10 µm in a 25 cm^2^‐area LDS layer using LR305 dye as the luminescent material. A zero *AR* corresponds to a planar LDS layer with a *DE* efficiency of 12%, as described in Section 2.3. Introducing micro‐texturing of any geometry, position, or orientation within an LDS layer more than doubles the *DE* efficiency compared with planar LDS layers. This enhancement occurs because surface texturing first alters the local surface normal, which modifies the conditions for TIR by increasing the critical angle (Figure S6). As a result, fewer photons satisfy the TIR condition, leading to increased light extraction (Figure 4). However, the maximum *DE* efficiency depends on the curvature characteristics of the micro‐structures, including the number of distinct (difference in angular range and orientation) surface normal and *AR*. Each structure exhibits a unique maximum *DE* efficiency under specific geometric conditions. Ranking these maxima from highest to lowest shows that bottom‐P Do achieve the highest *DE* (39%, *AR* = 1.0), followed by bottom‐P Co (37%, *AR* = 0.9) and bottom‐P Py (36%, *AR* = 0.9). slightly lower maxima are observed for top‐I and P Py (36%, *AR* = 0.45), followed by bottom‐I Cyl (34%, *AR* = 0.9), top‐I Pri (31%, *AR* = 0.45), and bottom‐P H‐Cyl (30%, *AR* = 0.9).

Key takeaways include that Do structures achieve the highest maximum *DE* efficiency. This is because the structures exhibit a continuous distribution of local surface normals that vary smoothly across the surface over a broad angular range. This distribution, combined with the isotropic (spherical) emission of the dye, produces a Lambertian angular distribution (Figure S7a,b) of *DE*‐converted red photons from a Do‐textured LDS layer. Pyramidal (Py) micro‐structures have higher maximum *DE* efficiency than prismatic (Pri) structures. This is because Py structures consist of four inclined planar facets that converge at an apex while Pri structures present two optically relevant planar facets, resulting in a greater number of discrete surface normal orientations. Although Do and H‐Cyl exhibit similar curvature, H‐Cyl provide fewer local surface normal orientations and therefore achieve lower maximum *DE* efficiency. Only the best‐performing orientation for each position is shown for conciseness. The complete dataset, including all combinations of orientation (indented, protruding) and location (top, bottom), appears in Figure S8a–f.

#### 3.1.2 Downward Outcoupling of Unconverted Photons (*UPO* Efficiency)

**FIGURE 5:**
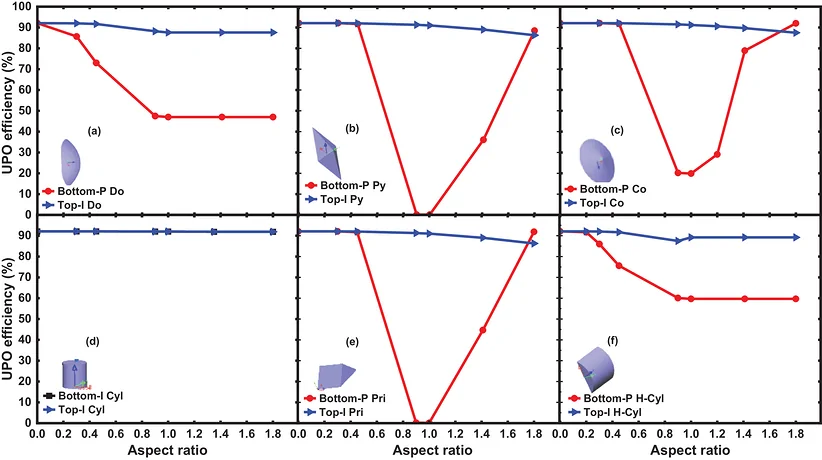
Unconverted photon outcoupling (*UPO*) efficiency of direct light as a function of aspect ratio (AR), surface position, and orientation for different micro‐structure geometries: (a) dome (Do), (b) pyramid (Py), (c) cone (Co), (d) cylinder (Cyl), (e) prism (Pri), and (f) half‐cylinder (H‐Cyl). Bottom‐P and top‐I denote bottom‐protruding and top‐indented micro‐structures, respectively.

As mentioned above, one objective of this work is to not only optimize how well the red luminescence is outcoupled, but also to maximize the *UPO* efficiency in the downward direction for photons that have not undergone absorption and emission. Maximizing the *UPO* efficiency in a downward direction is what ultimately matters when considering the full greenhouse design because it minimizes scattering, optical trapping, and retroreflection **losses** induced by the micro‐structures. Figure 5a–f illustrates the *UPO* efficiency of all photons (whole spectrum) from the bottom surface as a function of *AR*, position, and orientation for Do, Py, Co, Cyl, Pri, and H‐Cyl micro‐structures. Similar results occur for different orientations of the same micro‐structure on a given surface, indicating that the *UPO* efficiency for the I orientation closely matches that of the P orientation on the same surface (Figure S9a–f). Therefore, Figure 5a–f presents only one orientation per surface for conciseness. At an *AR* of zero (planar surfaces), all graphs show an *UPO* efficiency of 92%, with 8% Fresnel losses, corresponding to a reduction in transmitted irradiance of 1000 to 920 W m^−2^ as shown in Figure S10. As the *AR* increases beyond zero (indicating the presence of textures), the *UPO* efficiency decreases. This decrease is small when micro‐structures are located on the top surface (I or P), but becomes significant (depending on the type of micro‐structure) when they are positioned on the bottom surface (I or P), except in the case of bottom‐I Cyl layers.

As discussed previously, the presence of micro‐structures increases the number of distinct local surface normal. This change alters the angle of incidence from zero for normal incidence of unconverted light on a planar surface to greater non‐zero values. A non‐zero angle of incidence leads to a non‐zero angle of refraction. This causes a drop in intensity of the straight‐through rays (Figure S11a–f) due to change in direction of the extracted rays (Figure S12). Using top‐I Py layer an example, it is demonstrated that the angular direction of extraction of unconverted rays increases as the *AR* increases (Figure S13). This is an important relationship that will determine the number of the unconverted rays that reaches the greenhouse floor. For Do, Co, and H‐Cyl structures with planar regions between micro‐structures, straight‐through rays persist alongside rays extracted at non‐zero angles. Comparison of Figures S11 and S12 shows that straight‐through unconverted photons dominate the intensity distribution under broadband excitation, thereby masking the scattered component (Figure S12). Removing the straight‐through photons reveals the scattered component (Figure S12). For Do and H‐Cyl structures, which exhibit a continuous angular distribution of local surface normals, the scattered component forms a continuous loop, as expected.

The significant decrease in *UPO* efficiency when micro‐structures are positioned on the bottom surface arises from retroreflection losses. At a textured bottom interface, local surface normals can orient such that the angle of incidence exceeds the critical angle (∼42°). Because light propagates from a higher‐ to a lower‐refractive‐index medium, TIR occurs, reflecting rays back into the layer. For bottom‐P Py and bottom‐P Pri structures, when the geometry (*AR*) produces an incidence angle of approximately 45°, most rays reverse direction toward the source (retroreflection), as shown in Figure 5b,e. Retroreflection also occurs for bottom‐P Co structures (*AR* = 0.9–1) under normal incidence; however, the *UPO* efficiency does not approach zero (Figure 5c) because some rays transmit through planar regions between micro‐structures. Although the *UPO* efficiency for bottom‐I Py, Co, and Pri structures increases for *AR* > 1, the extraction angle also increases. This effect may cause unconverted rays from roof‐textured geometries to miss the greenhouse floor. For Do and H‐Cyl structures, which exhibit a continuous angular distribution of local surface normals, the *UPO* efficiency decreases from 92% (*AR* = 0) to approximately 50% (*AR* = 0.9) and then saturates. Increasing *AR* raises the fraction of local surface normals with angles equal to or greater than the critical angle (∼42°), thereby increasing the number of internally reflected rays. Beyond *AR* = 1, further increases do not significantly change this fraction, leading to saturation in *UPO* efficiency. The local surface normals of bottom‐I Cyl layers under normal irradiation exhibit orientations similar to those of planar surfaces (zero incidence angle); therefore, no significant change in extraction direction occurs, resulting in negligible variation in *UPO* efficiency (Figure 5d).

No retroreflection of unconverted photons occurs as the *AR* increases when micro‐structures are positioned on the top surface. At the top interface, light propagates from a lower‐refractive‐index medium (air) into a higher‐refractive‐index medium (*n* = 1.5), preventing TIR. Retroreflection, however, requires double TIR within the micro‐structure. Consequently, retroreflection of unconverted photons remains negligible for top‐textured configurations.

The limited occurrence of TIR for unconverted rays also explains the minimal variation in *UPO* efficiency observed for top‐positioned micro‐structures. This behavior indicates that top‐textured layers are suitable candidates for *REF* optimization in LDS greenhouse applications.

#### 3.1.3 Antagonistic Relationship Between Maximum *DE* Efficiency and *UPO* Efficiency

**FIGURE 6:**
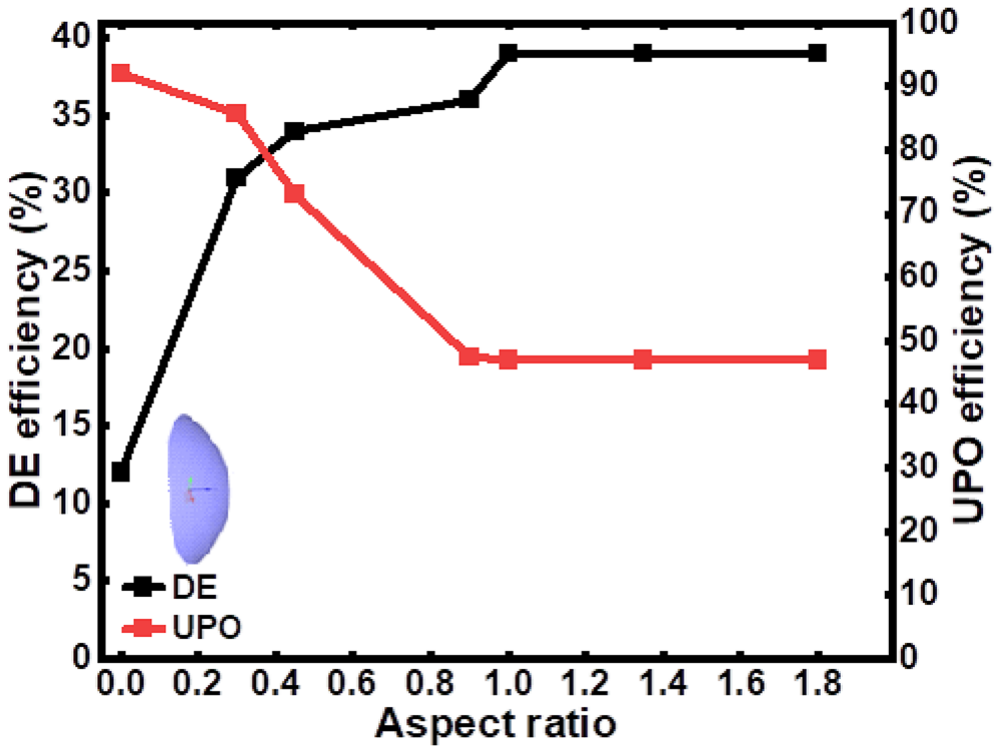
Downward extraction (*DE*) efficiency of converted red photons and Unconverted photon outcoupling (*UPO*) efficiency against aspect ratio for micro‐domes showing antagonistic relationship between the two dependent variables.

Simultaneously optimizing both *DE* and *UPO* efficiencies in small‐scale LDS layers is essential because these parameters govern the optical performance of full‐scale greenhouses. However, for most bottom‐positioned micro‐structures, except the bottom‐I Cyl configuration, increasing the *AR* to maximize *DE* efficiency reduces *UPO* efficiency. In other words, DE efficiency increases with increasing *AR*, whereas *UPO* efficiency decreases. Figure 6 illustrates this antagonistic relationship for micro‐domes. Initially, increasing *AR* produces a substantial increase in *DE* efficiency and a corresponding reduction in *UPO* efficiency, indicating that the optical conditions increasingly favor converted‐photon extraction at the expense of unconverted‐photon outcoupling. As *AR* increases further, the rate of *UPO* reduction decreases and gradually approaches a plateau, suggesting the presence of a critical *AR* threshold for balancing *DE* and *UPO* performance. Pyramids, cones, prisms, and half‐cylinders exhibit similar behavior. Cylinders, however, differ because the *UPO* efficiency of unconverted photons remains approximately constant as the *DE* efficiency of converted photons increases. This behavior demonstrates that geometrical conditions that enhance *DE* efficiency can simultaneously suppress *UPO* efficiency, thereby creating a trade‐off between the two performance metrics. Achieving simultaneous optimization of *DE* and *UPO* efficiencies therefore requires identifying the critical *AR* threshold at which both metrics remain favorable. This critical *AR* threshold likely occurs at relatively small aspect ratios for most geometries. For example, Figure 6 suggests that the balance point occurs at an *AR* less than or equal to 0.45. Identifying the *AR* range over which this balance occurs was critical for selecting suitable aspect ratios for the geometries examined in Section 3.1.4.

#### 3.1.4 Intensity Distribution of Luminescent Photons for Selected LDS Layers

**FIGURE 7:**
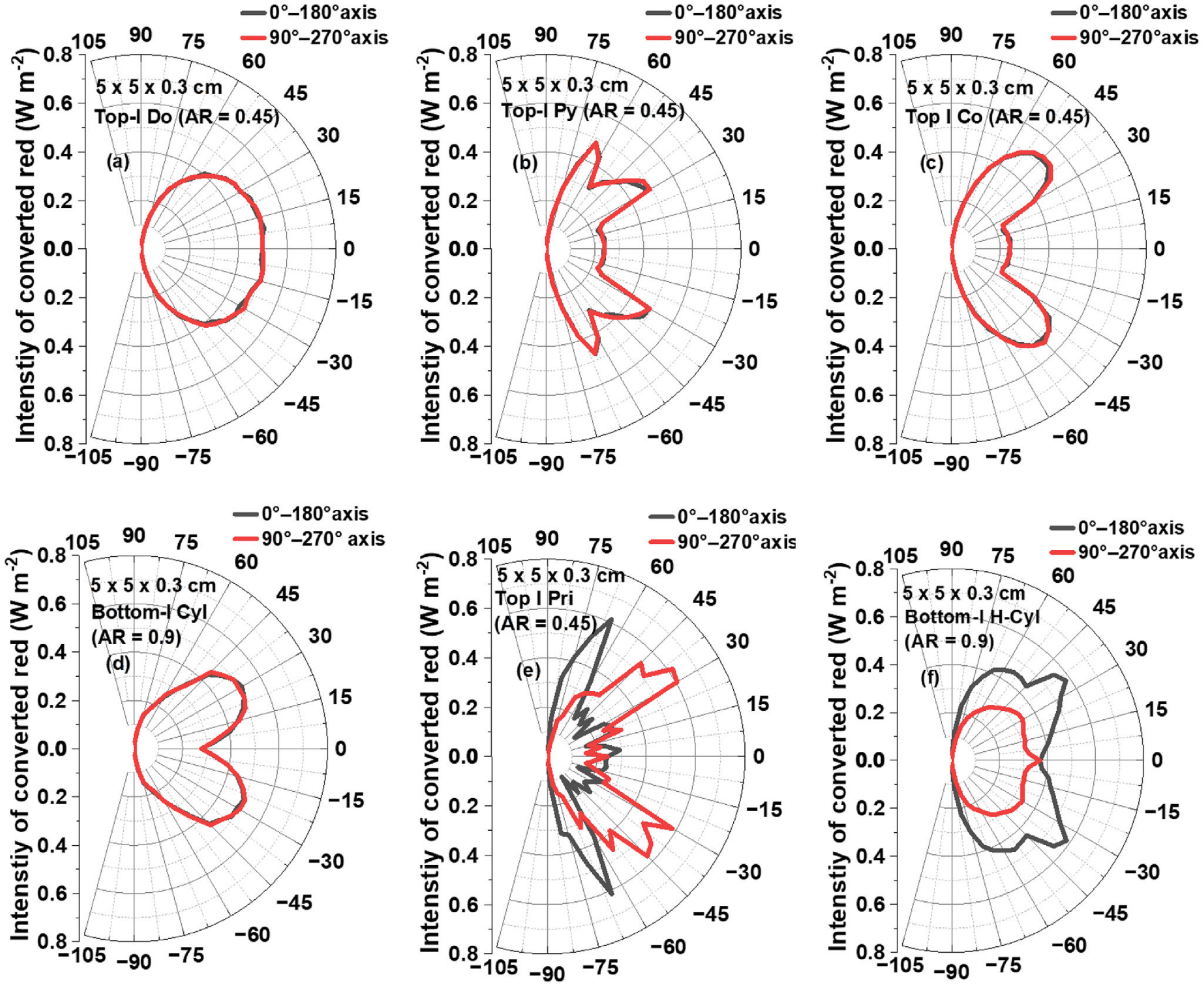
Angular intensity distribution of luminescent photons from luminescent downshifting (LDS) layers with (a) top‐indented (top‐I) dome (Do; *AR* = 0.45), (b) top‐indented (top‐I) pyramid (Py; *AR* = 0.45), (c) top‐indented (top‐I) cone (Co; *AR* = 0.45), (d) bottom‐indented (bottom‐I) cylinder (Cyl; *AR* = 0.9), (e) top‐indented (top‐I) prism (Pri; *AR* = 0.45), and (f) top‐indented (top‐I) half‐cylinder (H‐Cyl; *AR* = 0.3) micro‐structures. Normally incident monochromatic light at 577 nm (absorption peak) was used for excitation to minimise the dominant contribution of unconverted photons to the angular distributions of the luminescent photons.

As stated previously, the detectors for the small‐area LDS layers were placed 1 µm below the lower surface, thus effectively capturing light emitted at any angle. However, since the goal is to progress toward full‐scale greenhouse design, it is important to know exactly what angles the photons are emitted at. Figure 7a–f presents the angular intensity distributions of luminescent photons from selected LDS layers: top‐I Do (*AR* = 0.45), top‐I Py (*AR* = 0.45), top‐I Co (*AR* = 0.45), bottom‐I Cyl (*AR* = 0.9), top‐I Pri (*AR* = 0.45), and top‐P H‐Cyl (*AR* = 0.3), all doped with LR305 dye. These layers meet the optimal design criteria described in Sections 3.1.1 and 3.1.2, which prioritize high *DE* efficiency of converted red photons and high *UPO* efficiency (i.e., minimal trapping and retroreflection). The analysis focuses on the extraction directionality of converted red photons rather than absolute intensity because a single excitation wavelength (577 nm) was used to suppress the dominance of straight‐through unconverted photons.

Comparison with the intensity distributions of converted photons from a planar LDS layer (Figure S7a) and with those from the six textured layers simulated under identical conditions (Figure S14) shows a clear enhancement in luminescent extraction for all textures, consistent with the trends observed in Figure 4. In addition to the explanation provided in Section 3.1, separate simulations (Figure S6) demonstrated that textures of any shape increase the critical angle from 42° (for planar layers) to higher, shape‐dependent values (Figure S6a). The shape‐dependent transmitted irradiance profiles shown in Figure S6 explain the differences in the angular intensity distributions of converted photons in Figure 7a–f. Planar LDS layers exhibit a Lambertian extraction profile, consistent with isotropic emission from randomly oriented dye molecules superimposed on a single local surface normal perpendicular to the planar surface. In contrast, the textured LDS layers exhibit shape‐dependent angular extraction profiles. Among the tested geometries, only the Do yields a near‐isotropic distribution due to continuously varying surface normal in all directions (fully two‐dimensional continuous variation). The Py and Pri produce discrete lobed patterns corresponding to preferred extraction directions defined by their finite sets of surface normals. The Co and Cyl produce continuous but anisotropic angular distributions due to rotational symmetry of constant‐tilt surface normals (one‐dimensional continuous variation). The H‐Cyl exhibits a hybrid profile, characterized by an inner circular distribution and outer irregular lobes, resulting from the combination of a flat surface a curved surface with continuously varying normals in one direction. Profiles along the 0 – 180° axis for the Do, Py, Co, and Cyl structures coincide with those along the 90 – 270° axis, reflecting their symmetric properties. In contrast, the profiles for the Pri and H‐Cyl structures differ along these axes due to their anisotropic geometry and lack of equivalent symmetry in orthogonal directions.

A significant difference exists between the intensity distribution profiles of converted red photons (*DE* efficiency, Figure 7a–f) and those of unconverted photons (*UPO* efficiency, Figure S11) for the same micro‐structures. This difference confirms that the profiles strongly depend on the nature of the incident light, specifically, isotropic emissions for converted red photons and parallel incident rays for sunlight. Consequently, the intensity profiles of extracted light from an LDS layer depend strongly on both the nature of the incident light source and the geometry of the surface textures.

#### 3.1.5 Downward Extraction Efficiency of Converted Red versus Micro‐Structure Width, Spacing, and Layer Size

**FIGURE 8:**
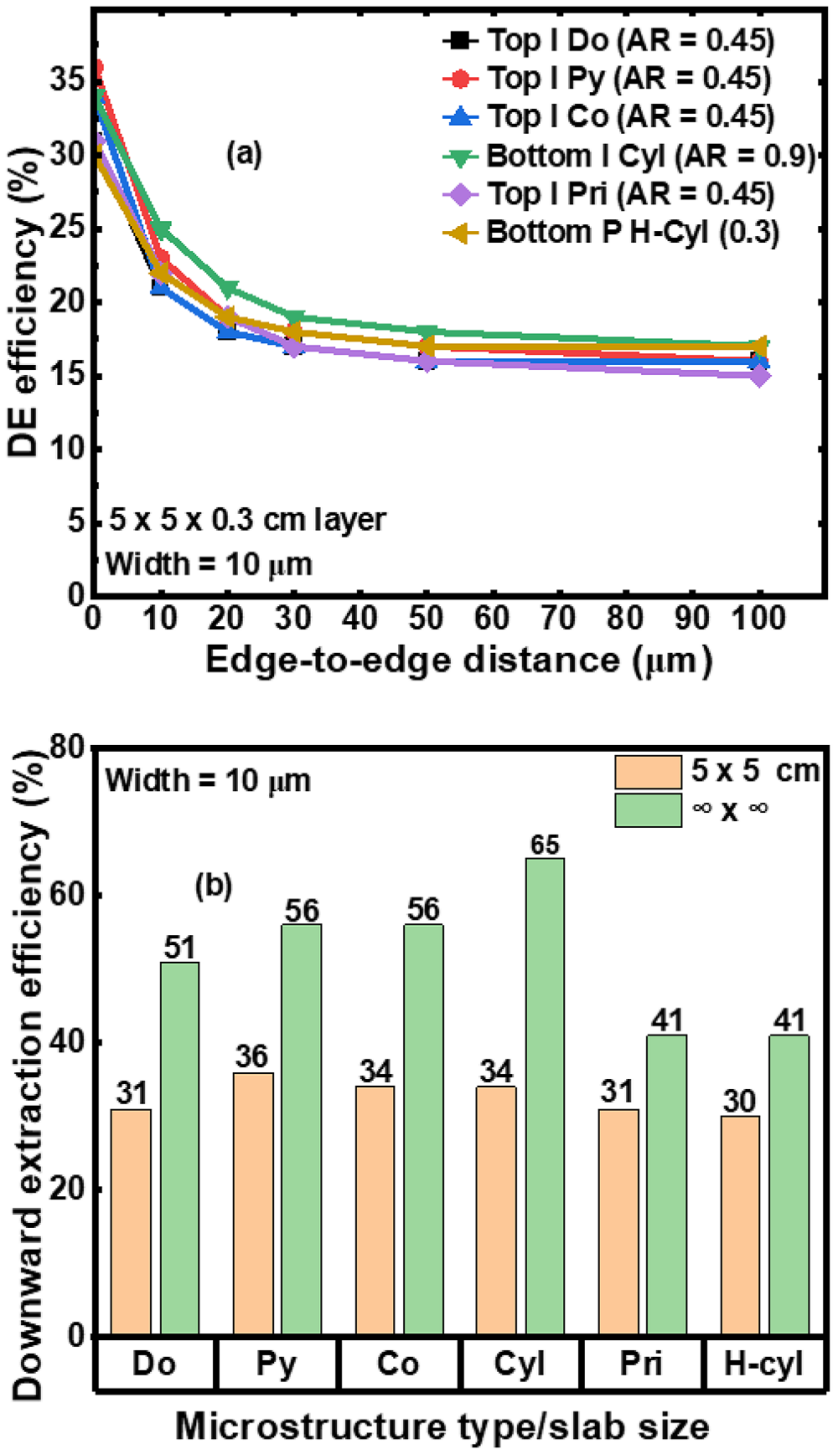
Dependence of downward extraction (*DE*) efficiency on (a) edge‐to‐edge (EED) spacing and (b) luminescent downshifting (LDS) slab size for the previously selected LDS layers with top‐indented (top‐I) dome (Do; *AR* = 0.45), top‐indented (top‐I) pyramid (Py; *AR* = 0.45), top‐indented (top‐I) cone (Co; *AR* = 0.45), bottom‐indented (bottom‐I) cylinder (Cyl; *AR* = 0.9), top‐indented (top‐I) prism (Pri; *AR* = 0.45), and top‐indented (top‐I) half‐cylinder (H‐Cyl; *AR* = 0.3) micro‐structures using Lumogen F Red 305 (LR305) dye.

Since enhancing *DE* efficiency is a primary objective of this study, it is essential to understand how geometric design parameters govern the probability of luminescent photons interacting with micro‐structures and escaping through the bottom surface before being lost to waveguiding or slab‐edge escape. Figure 8 illustrates how micro‐structure edge‐to‐edge spacing and LDS slab size influence the *DE* efficiency of converted red photons for selected LDS layers: top‐I Do (*AR* = 0.45), top‐I Py (*AR* = 0.45), top‐I Co (*AR* = 0.45), bottom‐I Cyl (*AR* = 0.9), top‐I Pri (*AR* = 0.45), and top‐I H‐Cyl (*AR* = 0.3), all doped with LR305 dye. The micro‐structure width was set to 10 µm. Smaller micro‐structures reduce soiling risk while maintaining optical performance [24]. *DE* efficiency is highest when the edge‐to‐edge spacing is zero (i.e., when micro‐structures are in contact) and decreases exponentially as spacing increases (Figure 8a). This exponential decline reflects the reduced number density of micro‐structures and is consistent with the findings of Xu et al. (2022) [22]. Greenhouse glazing typically uses sheets larger than 100 × 100 cm; therefore, the effect of slab size was evaluated. The model represents this condition by placing perfectly reflecting mirrors at the edges of the LDS layers, effectively simulating an infinite (>100 × 100 cm) slab and increasing *DE* efficiency. As shown in Figure 8b, *DE* efficiencies for top‐I Do (*AR* = 0.45), top‐I Py (*AR* = 0.45), top‐I Co (*AR* = 0.45), bottom‐I Cyl (*AR* = 0.9), top‐I Pri (*AR* = 0.45), and top‐I H‐Cyl (*AR* = 0.3) increase from 31% to 51%, 36% to 56%, 34% to 56%, 34% to 65%, 31% to 41%, and 30% to 41%, respectively, when mirrors are added to the edges of 5 × 5 × 0.3 cm slabs to approximate ∞ × ∞ × 0.3 cm slabs. This trend is consistent with the known dependence of *DE* efficiency on device area due to reduced edge losses.

As shown in Figure S15A, *DE* efficiency increases rapidly at small feature widths before approaching a plateau. Above approximately 5 µm, increasing the width to 100 µm produces no meaningful change in *DE* for structures with the same geometry and *AR*. This behavior is consistent with geometrical optics because geometrically similar structures with a fixed *AR* preserve the local surface slopes and interface orientations that govern refraction and internal reflection [40], resulting in comparable extraction conditions despite differences in absolute size. Approximately 5 µm is therefore considered the practical lower feature‐width limit for the geometrical‐optics analysis in this study. Over the relevant visible wavelength range (∼0.4–0.7 µm), a 5 µm feature is approximately 7–12 wavelengths across, supporting a ray‐optical description of the dominant interface interactions.

The 3‐mm LDS layer was adopted as the baseline thickness because this thickness is suitable for incorporating the proposed surface micro‐structures. Its practical feasibility was demonstrated experimentally in the present study through the successful fabrication of a 5 × 5 × 0.3 cm LR305‐containing textured LDS sample, as described in Section S5.1. The feasibility of millimeter‐scale luminescent polymer plates is also supported by previous experimental studies reporting 3‐mm‐thick luminescent plates [41].

While the 3‐mm LDS layer remains the baseline structural configuration in this study, thinner layers were investigated (Figure S15B) to assess the extension of the micro‐textured LDS concept to flexible foil‐based greenhouse covers. Such configurations are relevant to precision roll‐to‐roll micro‐texturing [42], for which polymer foils with thicknesses up to approximately 400 µm are suitable for high‐fidelity pattern replication [43]. Using the fabrication approach described in Section S5.1, the LDS layer thickness can be reduced by decreasing the height of the confining frame while retaining the same surface‐texturing process. Figure S15B shows that *DE* increases as the layer thickness decreases from 3 mm and approaches a plateau below approximately 100 µm, consistent with the thickness‐dependent behavior reported by Xu et al. [22]. This improvement results from shorter propagation distances for internally trapped photons, which increase their likelihood of interacting with the textured interface and being redirected into the escape cone. However, reducing the layer thickness also shortens the optical path through the luminophore‐containing matrix and may therefore decrease photon absorption at a fixed luminophore concentration [44], consistent with the Beer–Lambert law. Increasing the luminophore concentration could compensate for this reduced absorption, although excessive concentrations may introduce concentration‐dependent losses such as self‐absorption or photoluminescence quenching. Thus, reducing the LDS layer thickness requires optimization of both layer thickness and luminophore concentration to balance photon absorption and extraction efficiency in thin‐film and foil‐based configurations.

### 3.2 Full‐Scale LDS Greenhouse Simulations

#### 3.2.1 Greenhouse with Sun Directly Overhead

To provide a reference framework for interpreting LDS greenhouse performance under realistic solar irradiation conditions (i.e., solar altitudes below 90°; Figure S17), this section investigates how textured LDS roof geometries influence the delivery of converted red photons and unconverted photons to the greenhouse floor under idealized overhead solar illumination (i.e., when the sun is normal to the greenhouse floor and the solar altitude is 90°). Under the idealised reference condition, 92% of the unconverted light incident on the roof of a transparent planar (T‐Pl) greenhouse transmits directly to the floor (Figure S16), corresponding to an average irradiance (*I*_ave_) of 920 W m^−2^.

This study optimizes the total number of converted and unconverted red photons reaching the greenhouse floor, which is proportional to *I*_ave_. The selected textured LDS geometries (Section 3.1.4) with the given *AR*s reduce *I*_ave_, so lower *AR*s were investigated (Table S1). The results show that *AR* = 0.2 yields the highest *I*_ave_ (i.e., minimal scattering losses) across all geometries. At *AR* = 0.2, unconverted rays reach the greenhouse floor at angles close to 0° (Figure S18a–f). As a result, only a small fraction of the rays escape through the greenhouse walls before reaching the floor. In particular, top‐I Do (*AR* = 0.2) and bottom‐I Cyl (*AR* = 0.2) exhibit the strongest straight‐through components, indicating the highest average irradiance at the greenhouse floor. This behavior also explains why the intensity distribution of converted red photons at the floor of a planar LDS greenhouse (Figure S19a), where the detector is positioned more than 2 m below the roof, is less intense than the intensity distribution measured directly beneath a planar LDS layer (Figure S14a). In the greenhouse configuration, photons propagate over much longer distances and a larger fraction of photons is lost through the sidewalls before reaching the detector. The same effect explains the directional differences in photon enhancement observed between the LDS layers and the greenhouse configurations. For the LDS layers (Figure S14b–f), where the detector is positioned approximately 1 µm below the bottom surface, the enhancement profiles along the 0 – 180° and 90 – 270° directions overlap because photon escape losses remain negligible. In contrast, the greenhouse configurations exhibit stronger enhancement along the east–west direction than along the north–south direction (Figure S19b–g). This asymmetry arises from the greenhouse geometry because the roofline runs along the east–west axis and spans 10 m, whereas the north–south axis spans only 4 m. Consequently, photons traveling along the shorter north–south direction are more likely to escape through the sidewalls before reaching the greenhouse floor.

As defined previously (Equation S4), the *REF* represents the percentage increase in the total number of red photons (both converted and unconverted) reaching an LDS greenhouse relative to the number of red photons available in a T‐Pl or dye‐free planar greenhouse (natural red photons) of the same dimensions. The study then evaluates *REF* for geometries with *AR* = 0.2 that meet the *I*_ave_ selection criterion (Table 1). The results identify top‐I Do (*AR* = 0.2) and bottom‐I Cyl (*AR* = 0.2) as the optimal configurations. These geometries are used for the remainder of the study. Under this definition, the *REF* of a T‐Pl greenhouse is 0%. When the sun is directly overhead, a planar LDS greenhouse doped with LR305 achieves an *REF* of 5%, whereas doping with GloGro yields an *REF* of 2% (Table 1). The higher *REF* for LR305 arises because it absorbs in the green region of the electromagnetic spectrum, which contains a greater photon flux than the UV region where GloGro absorbs. For textured configurations under the same conditions, top‐I Do (*AR* = 0.2) and bottom‐I Cyl (*AR* = 0.2) achieve *REF* values of 9% and 10%, respectively, with LR305, compared with 2% and 3%, respectively, with GloGro. Micro‐structures therefore produce a significant increase in *REF* when combined with LR305 but only a minor increase when combined with GloGro, due to the higher number of converted photons generated by LR305.

**TABLE 1:** Comparison of red enhancement fraction (*REF*) at the floor of the model luminescent downshifting (LDS) greenhouse for Lumogen F Red 305 (LR305) dye and GloGro luminophore with different roof micro‐structure geometries, textured surfaces, and orientations when the sun is normal to the greenhouse floor. The investigated configurations include planar, top‐indented (top‐I) dome (Do), pyramid (Py), cone (Co), prism (Pri), bottom‐indented (bottom‐I) cylinder (Cyl), and top‐protruding (top‐P) half‐cylinder (H‐Cyl) micro‐structures.

Micro‐structure geometry, textured surface, and orientation	Aspect ratio	*REF* at floor level for LR305 dye [%]	*REF* at floor level for GloGro [%]
Planar	0.00	5	2
Top‐I Do	0.20	9	2
Top‐I Py	0.20	5	−1
Top‐I Co	0.20	5	−1
Bottom‐I Cyl	0.20	10	3
Top‐I Pri	0.20	4	0
Top‐P H‐Cyl	0.20	7	1

#### 3.2.2 Greenhouse Under Realistic Solar Irradiation in Karlsruhe

##### 3.2.2.1 Average unconverted irradiance at greenhouse floor

**FIGURE 9:**
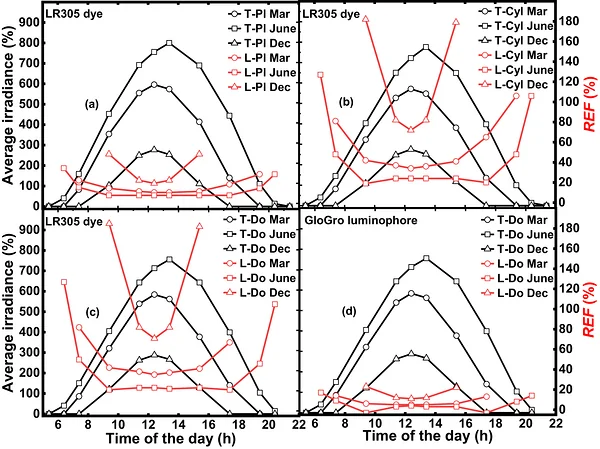
Average floor‐level irradiance and red enhancement fraction (*REF*) for model greenhouses using LR305 dye are shown for (a) transparent planar (T‐Pl) and luminescent planar (L‐Pl), (b) transparent dome (T‐Do) and luminescent dome (L‐Do), and (c) transparent cylinder (T‐Cyl) and luminescent cylinder (L‐Cyl). Panel (d) shows results for T‐Do and L‐Do using GloGro luminophore. All data are plotted as a function of time of day on March 21, June 21, and December 21.

Because realistic solar elevations introduce additional optical losses, including increased Fresnel losses, ray escape through greenhouse sidewalls, and scattering or trapping of unconverted photons, this section investigates how seasonal changes in solar elevation influence average irradiance at the greenhouse floor for planar and textured LDS greenhouse configurations (Figure 9, **black curves**). In Karlsruhe, the maximum solar elevation varies throughout the year (Figure S17a), reaching a maximum of 64.4° on June 21 (summer solstice) and a minimum of 17.5° on December 21 (winter solstice). On the spring and autumn equinoxes (March 21 and September 21), the maximum solar elevation is 41.4°. Because the spring and autumn equinoxes share the same solar elevation, this study considers three representative days (March 21, June 21, and December 21) to capture seasonal variation.

At a solar elevation of 64.4°, optical pathways shift from normal (when sun is directly overhead) to oblique incidence relative to the greenhouse floor. Some light reaches the floor through the south vertical wall, whereas another portion transmits through the roof at a slight southward inclination. For the fraction transmitted through the walls, the incidence angle is 64.4°, resulting in an estimated Fresnel loss of approximately 22%. For the portion transmitted through the roof, which slopes at 20°, the incidence angle is about 46°, corresponding to a Fresnel loss of approximately 10%. Depending on the relative fractions of light entering through the wall and roof, overall Fresnel losses range from roughly 16% to 22%. At this elevation, unconverted rays reaching the floor of the modelled greenhouse propagate at an angle of about +25° and −25° from vertical (Figure S20b). As a result, some rays that would reach the floor under normal incidence escape through the north and south walls. The combined effects of increased Fresnel losses and ray inclination reduce average irradiance to approximately 800 W m^−2^ at solar noon on June 21 (13:24). As shown in Figure 9a, the number of lost rays increases at lower solar elevations, such as such as those observed on March 21 and December 21, leading to the observed decrease in maximum average irradiance. Seasonal changes in the solar trajectory (changes in the solar azimuth angle; Figure S17b), including the shift in sunrise and sunset positions between summer and winter, also contribute to the observed average irradiance variations. For instance, during winter, the sun rises and sets farther toward the southeast and southwest, respectively, whereas in summer it shifts toward the northeast and northwest. Average irradiance is also generally lower near sunrise and sunset due to low solar altitudes.

Texturing reduces the maximum average irradiance from 800 W m^−2^ for the planar greenhouse to 756 W m^−2^ for top‐I Do (*AR* = 0.2) and 776 W m^−2^ for bottom‐I Cyl (*AR* = 0.2) at peak solar irradiance (solar noon on June 21). This reduction likely results from small scattering and trapping losses, as discussed in Section 3.1.2. Under these conditions, top‐I Do scatters unconverted light slightly more than bottom‐I Cyl. This trend is consistent with the LDS layer results reported in Section 3.1.2.

##### 3.2.2.2 Red Enhancement Fraction (REF) at floor level

**FIGURE 10:**
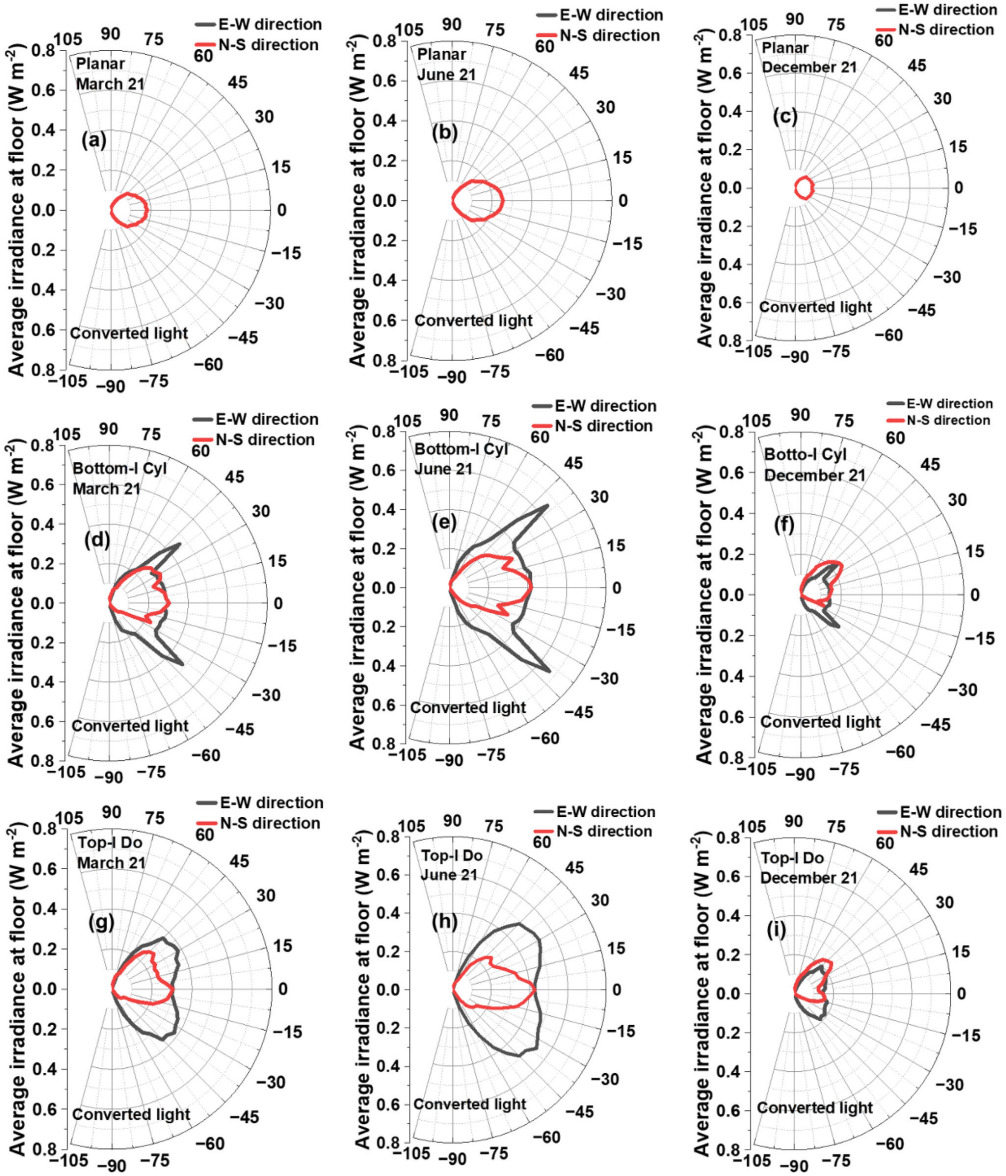
Intensity distribution of converted light at the floor of a model luminescent downshifting (LDS) greenhouse for (a–c) planar, (d–f) top‐indented (top‐I) dome (Do; *AR* = 0.2), and (g–i) bottom‐indented (bottom‐I) cylinder (Cyl; *AR* = 0.2) roofs using Lumogen F Red 305 (LR305) dye on March 21 (12:24), June 21 (13:24), and December 21 (12:24) at solar noon in Karlsruhe, Germany. The greenhouse measures 10 m along the east–west (E–W) direction (longer side) and 4 m along the north–south (N–S) direction (shorter side).

This section examines the temporal variation of floor‐level *REF* in model greenhouses incorporating LDS layers (Figure 9a–d, red curves). The analysis compares dye‐free or transparent planar (T‐Pl) and luminescent configurations across planar, dome, and cylindrical geometries doped with LR305 dye, as well as dome structures doped with GloGro. In a planar LDS greenhouse, *REF* is generally slightly higher under real‐world solar irradiation (this section) than in idealized conditions when the sun is directly overhead (Section 3.2.1), due to the smaller number of natural red photons available (*N*_red,  std_) at low solar altitudes. This also explains why all *REF* values are greater at sunrise and sunset compared with solar noon (Figure 9, red curves). Seasonal trends further highlight this effect: Throughout daytime hours (9:00 – 17:00), December exhibits the highest *REF*, followed by March, and June, reflecting the corresponding sequence of peak solar altitudes (Figure S17a). At low solar altitudes, sunlight predominantly strikes vertical walls rather than the roof, which reduces the number of natural red photons reaching the greenhouse floor. However, the broad angular distribution of extracted photons (Figure 10) ensures that converted red light still reaches the floor, contributing to PAR throughout the greenhouse.

The introduction of top‐I Do (*AR* = 0.2) and bottom‐I Cyl (*AR* = 0.2) micro‐structures significantly increases *REF* across all seasons and times of day. This effect is evident in Figure 9, where the red curves in panels (b) and (c) show higher *REF* values than those in panel (a). These results demonstrate that micro‐texturing of LDS greenhouses enhances the red photon flux available for photosynthesis. Previous studies have shown that increased red photon flux accelerates plant growth and enhances biomass production [11, 45, 46, 47], indicating strong potential for improved crop yield. This enhancement is quantitatively evident. For greenhouses using LR305 dye at solar noon, top‐I Do increases *REF* from 11% to 36% on March 21, from 8% to 23% on June 21, and from 20% to 72% on December 21. Similarly, bottom‐I Cyl increases *REF* from 11% to 34% on March 21, from 8% to 24% on June 21, and from 20% to 72% on December 21. In contrast, top‐I Do greenhouses using GloGro (Figure 9d) exhibit lower *REF* values than planar greenhouses using LR305 (Figure 9a), consistent with the previously discussed absorption characteristics. Note that all the previous results are based direct solar irradiation.

To assess the effect of diffuse illumination on *REF*, an additional simulation was conducted for June 21 under 100% diffuse irradiance of 104 W m^−2^ but using the same spectral profile. This analysis is relevant to European conditions, where diffuse radiation can constitute a substantial fraction of the incident solar irradiance [48]. Under 100% diffuse illumination, the calculated *REF* was 24%, only one percentage point higher than the corresponding value of 23% obtained under direct illumination on June 21. This small difference indicates that *REF* remains relatively insensitive to the change from direct to fully diffuse illumination under the simulated conditions. However, the angular distribution of the unconverted photons changes substantially: direct illumination produces a pronounced straight‐through component, whereas fully diffuse illumination results in a broader, near‐Lambertian distribution (Figure S20B).

These results reveal a clear seasonal trend: the relative enhancement is greatest during periods of low natural red light, such as winter, where *REF* nearly quadruples. Spring and summer also experience substantial gains, although to a lesser extent. From a crop growth perspective, these enhancements translate to higher red‐light availability during critical photosynthetic periods, promoting more uniform canopy illumination, increasing LUE [12], accelerating biomass accumulation, and ultimately improving yield. Greenhouse designs incorporating optimized micro‐structures can therefore mitigate seasonal light limitations, ensuring crops maintain high productivity even under low natural red‐light conditions.

##### 3.2.2.3 Angular and Spatial Distribution of Converted and Unconverted Light at Floor Level

**FIGURE 11:**
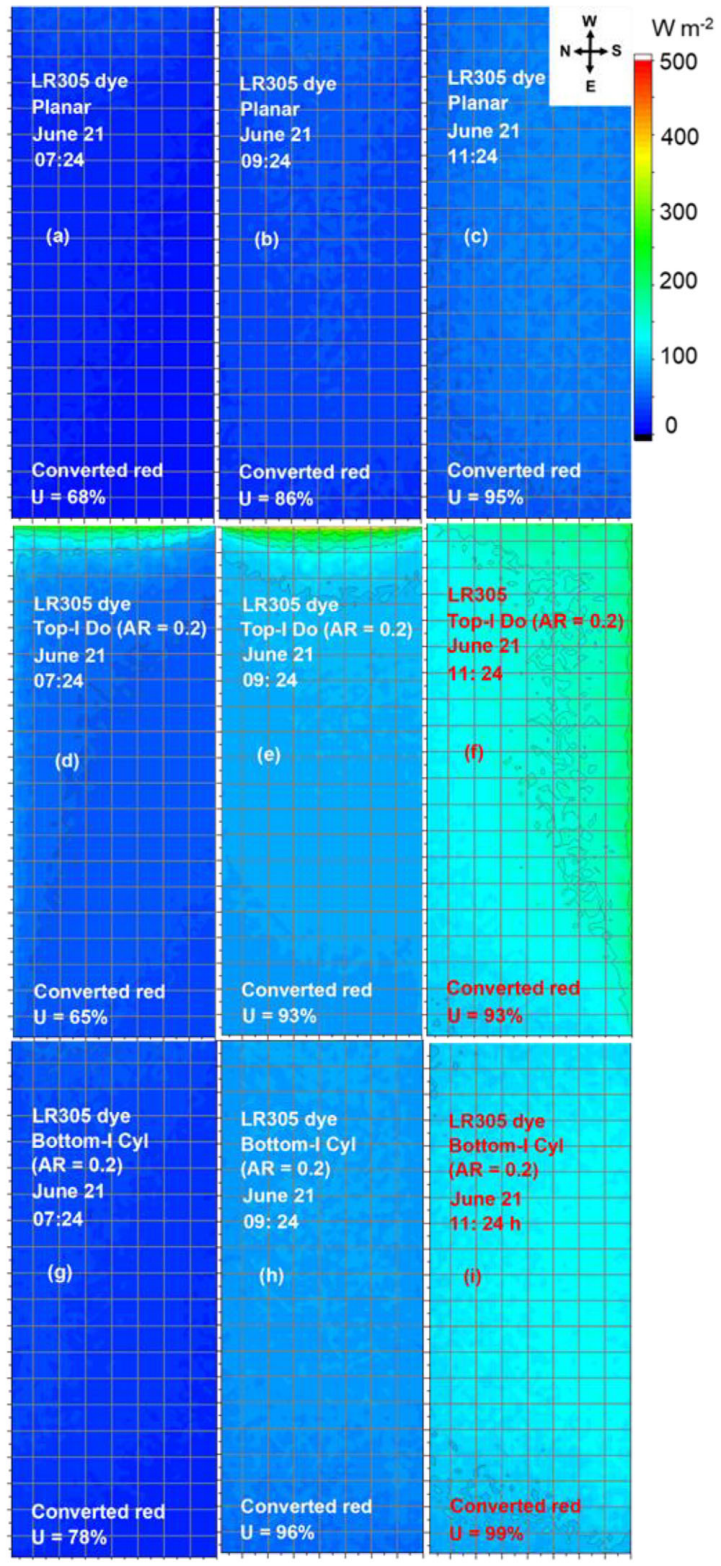
Intensity maps of converted red photons at the 10 m (east–west; E–W) × 4 m (north–south; N–S) floor of a model luminescent downshifting (LDS) greenhouse for (a–c) planar, (d–f) top‐indented (top‐I) dome (Do; *AR* = 0.2), and (g–i) bottom‐indented (bottom‐I) cylinder (Cyl; *AR* = 0.2) roofs on June 21 at three times of day: early morning (07:24), mid‐morning (09:24), and late morning (11:24) in Karlsruhe, Germany.

This section examines the angular distribution across seasons (spring, summer, and winter) in Karlsruhe, as shown in Figure 10. It also analyses the spatial distribution of converted red photons at different times of day (early morning, mid‐morning, and late morning), as illustrated in Figure 11a–i. A uniform spatial distribution of converted photons ensures that all plants within the greenhouse receive similar light intensity, thereby promoting uniform crop growth enhancement [46, 47]. Monsi and Saeki demonstrated that plant productivity depends on factors such as light intensity, photosynthetic activity, and the amount of assimilatory tissue. The figure of merit used to quantify the spatial light distribution within the greenhouse is the uniformity (*U*). The method used to determine *U* in this work follows that reported by Matsumori et al. [39]. The spatial distributions in Figure 11 show that the *U* of converted red photons increases from early morning to late morning. Bottom‐I Cyl (*AR* = 0.2) improves *U* more significantly than top‐I Do (*AR* = 0.2).

The angular intensity distributions (Figure 10) demonstrate that the micro‐structure geometry modifies the propagation angles of extracted luminescence, thereby influencing both light penetration into the crop canopy [49] and lateral (spatial) redistribution (*U*) within the greenhouse. Light penetration is an important determinant of the within‐canopy light environment because it affects photosynthetic acclimation, resource allocation, and plant responses to spatial variations in irradiance [49]. As shown in Figures S18a–f and S20a–l, the unconverted component remains predominantly directional, whereas the converted luminescence from the LDS greenhouses exhibits a substantially broader angular distribution (Figure 10a–c and Figure S19a). Surface micro‐texturing further modifies this diffuse component by redistributing the extracted luminescence over different propagation angles. From a greenhouse‐design perspective, however, the optimum distribution is not necessarily purely directional or perfectly Lambertian. For roof‐mounted LDS layers, a narrowly downward‐directed distribution would maximize photon delivery to the greenhouse floor by limiting sidewall losses, whereas a broad, near‐Lambertian downward distribution provides greater angular diversity and spatial coverage for illuminating differently oriented leaves within the crop canopy. A favorable design target for the present roof geometry is therefore a broad downward distribution weighted toward smaller propagation angles, providing a compromise between efficient photon delivery and angular redistribution. Among the investigated micro‐structures, the top‐I Do (*AR* = 0.2) most closely approach this behavior, combining broad, near‐Lambertian redistribution with high greenhouse‐level *REF*. The optimum angular profile is nevertheless dependent on the position of the LDS surface. For LDS layers incorporated into the greenhouse sidewalls, for example, Lambertian emission could direct a substantial fraction of the extracted luminescence towards the opposite wall rather than the crop. In such configurations, asymmetric micro‐structures, such as sawtooth‐like textures, designed to preferentially redirect luminescence downwards toward the crop canopy may provide a more appropriate angular distribution.

When the solar altitude deviates from 90°, as shown for different dates in Figure 10, the angular distribution patterns of converted intensity along the north–south floor profile coincide with those along the east–west direction and remain symmetric about 0° only in planar greenhouses and for textured greenhouse at solar noon. In textured greenhouses at sunrise and sunset, the symmetry axis of the north–south profile rotates as solar altitude decreases. For example, at a solar altitude of 64.4° on June 21, the profile rotates by approximately 0°. At 41.4° on March 21, the rotation increases to about 15°, and at 17.2° on December 21, it reaches approximately 30°. This dependence on solar altitude is consistent with the trend observed for the *U* in Figure 11a–i. The rotation of the symmetry axis may enhance light distribution and consequently improve *U*, as textured greenhouses exhibit greater uniformity than planar configurations. Uniformity also depends strongly on solar altitude. For example, in the idealized reference scenario in which the sun is positioned directly overhead, *U* approaches 100% for both planar and textured greenhouses (Figure S21a‐g). However, under realistic conditions, *U* decreases as the solar elevation angle decreases (Figure S22a–i). The angular intensity distribution along the north–south direction in textured LDS greenhouses is lower than that along the east–west direction (Figure 10 and Figure S19a–g). In contrast, the angular intensity distribution along the 0°–180° axis for textured LDS layers, measured with detectors positioned 1 µm below the bottom surface, coincides with that along the 90°–270° axis (Figure 7 and Figure S14). This difference arises because the greenhouse width (4 m) is smaller than its length (10 m). As a result, photons extracted at higher angles escape through the side walls in the north–south direction and do not reach the floor. Increasing the greenhouse width can reduce this loss and improve photon collection at floor level.

Overall, these findings suggest that optimizing both micro‐structure design and greenhouse geometry (particularly increasing greenhouse width to improve *REF*) can enhance light uniformity and intensity. Such improvements are critical for maximizing photosynthetic efficiency, promoting uniform crop growth, and increasing overall greenhouse productivity across seasons.

## 4 Conclusions and Recommendations for Future Work

This study used Monte Carlo ray tracing in LightTools to optimize micro‐textured luminescent downshifting (LDS) layers and their integration into realistic horticultural greenhouses. It linked the effects of micro‐structuring (including enhanced downward outcoupling of converted red photons, scattering and trapping of unconverted photons, retroreflection within LDS layers, and photon losses through greenhouse walls) to key performance metrics, including: i) the downward extraction (*DE*) efficiency of luminescence; ii) the unconverted photon outcoupling (*UPO*) efficiency; iii) the average floor‐level irradiance; and iv) the red enhancement fraction (*REF*).

The study examined six micro‐structures (i.e. domes (Do), cones (Co), pyramids (Py), cylinders (Cyl), half‐cylinders (H‐Cyl), and prisms (Pr) and how the shape, aspect ratio, width, density, orientation, and placement of the structures influenced *DE* efficiency in LDS layers doped with either LR305 or GloGro luminophore. The results showed that micro‐texturing more than doubled *DE* efficiency, increasing it from 12% for planar 5 × 5 × 0.3 cm LDS layers to a maximum of 39% for bottom‐protruding dome structures (aspect ratio; *AR* = 1.0), demonstrating the strong effectiveness of surface texturing in enhancing converted photon outcoupling. *UPO* efficiency remained high (approximately 92%) for planar and top‐textured LDS layers but decreased significantly for bottom‐textured configurations due to increased scattering, retroreflection, and optical trapping. An inherent antagonistic relationship between maximizing *DE* efficiency of converted photons and preserving the transmittance of unconverted photons is revealed. Bottom‐indented cylindrical 5 × 5 × 0.3 cm LDS layers provided an optimal balance between converted and unconverted photon outcoupling, achieving a *DE* efficiency of 34% (*AR* = 0.9) while minimizing retroreflection losses. Furthermore, when edge reflectors approximated infinitely large layers, bottom‐indented cylindrical structures achieved a maximum *DE* efficiency of 65% with LR305, underscoring the critical role of layer size in maximizing downward converted photon extraction.

In addition, it simulated the performance of large‐scale LDS greenhouses under real‐world solar irradiation conditions in Karlsruhe, predicting daily and seasonal variations in *REF*, average irradiance of unconverted photons, and the spatial and angular distributions of converted red light at floor level. At the greenhouse scale, micro‐textured LDS systems achieved only modest gains in *REF* under direct overhead solar illumination, increasing from 5% for planar LR305 systems to 10% for optimized textured configurations. Under realistic solar elevations, increased Fresnel reflection and angular escape through greenhouse walls significantly reduced floor‐level irradiance compared with ideal overhead conditions, while micro‐texturing introduced only minor additional losses for optimized top‐indented dome (top‐I Do) and bottom‐indented cylinder (bottom‐I Cyl) configurations, each with an *AR* of 0.2. *REF* increased as solar altitude decreased, driven by reduced availability of natural red photons and sustained delivery of converted photons due to their broad angular distribution, identifying solar elevation and photon distribution as dominant factors governing *REF*. Micro‐texturing amplified this trend, with optimized geometries (top‐I Do and bottom‐I Cyl, *AR* = 0.2) consistently enhancing *REF* across all times of day and seasons (tripling *REF* in summer, increasing it more than threefold in spring, and achieving more than a fivefold increase in winter) particularly under low solar altitudes where the ratio of converted to natural red photons was highest. Micro‐texturing also rotated the angular distribution of converted photons away from the north–south direction at sunrise and sunset, enhancing spatial redistribution of light and significantly improving uniformity, especially under low solar elevations. These results showed that optimal LDS layer performance did not directly translate to greenhouse‐scale efficiency, highlighting the need for integrated optimization of both LDS layers and greenhouse design.

Future work should build on the preliminary design rule identified in this study by prioritizing smooth, continuously curved, non‐apical micro‐structures and optimizing their parameters (*AR*, width, density, orientation, and placement) to balance photon extraction and angular redistribution. This design rule for the greenhouse configuration investigated is supported by the comparative performance of the six investigated geometries, particularly the higher *REF* and broad, near‐Lambertian angular distributions achieved by the Do and Cyl. Further investigation of truncated cones and pyramids could help isolate the effects of apices and inclined surfaces, while rounded or smoothed variants of these geometries could clarify whether replacing flat facets and sharp transitions with continuous curvature further enhances photon extraction and redistribution. Simultaneous texturing of the top and bottom LDS surfaces with complementary geometries could provide additional control over photon extraction and spatial light distribution at the crop level. Further advances could also be obtained through spectral engineering of the luminophore. Specifically, future work could examine a single multifunctional luminophore that has complementary, selective absorption bands in the UV (300–400 nm) and green (500–600 nm) spectral ranges, while reducing absorption of photosynthetically important blue radiation and producing emission in the blue and red spectral regions. This spectral selectivity would address a drawback of broadly absorbing luminophores such as LR305, which are able to convert a relatively large portion of the incident spectrum but can also absorb some of the beneficial blue radiation. A multifunctional luminophore could instead downshift UV photons to blue and/or red wavelengths and selectively convert a fraction of green photons into red light while better preserving the incident blue spectrum. Together with a high photoluminescence quantum yield (PLQY) and sufficiently large Stokes shifts, this dual‐absorption, dual‐emission approach could support more efficient and controlled spectral redistribution and offer increased flexibility for tailoring the transmitted spectrum to crop‐specific photosynthetic needs. Beyond optical and spectral optimization, future work should also investigate thin, flexible micro‐textured LDS foils compatible with continuous roll‐to‐roll fabrication, which has demonstrated the potential for structuring polymer films over square‐meter areas. Such an approach could facilitate scalable LDS integration into flexible greenhouse coverings, including polytunnels, while requiring simultaneous optimization of foil thickness, micro‐structure fidelity, luminophore concentration, and mechanical properties. Future research should also broaden the diffuse‐light analysis to cover different seasons, solar elevations, and realistic combinations of direct and diffuse irradiance that reflect greenhouse operating conditions. These simulations should systematically test how the diffuse fraction influences *DE*, *UPO*, *REF*, irradiance at floor level, and both spatial and angular light uniformity for each micro‐structure geometry. This expanded assessment will determine whether changes in the diffuse fraction affect the relative performance and the ranking of the micro‐structures, as observed under mostly direct illumination.

## Conflicts of Interest

The authors declare no conflicts of interest.

## Supporting information




**Supporting File 1**: gch270163‐sup‐0001‐SuppMat.docx.

**Supporting File 2**: gch270163‐sup‐0002‐SuppMat.zip.

## Data Availability

The data that support the findings of this study are available on request from the corresponding author. The data are not publicly available due to privacy or ethical restrictions.
