# Supplementary material for: Influence of Micro‐Scale Surface Texturing on Light Distribution From Luminescent Downshifting Layers for Next‐Generation Greenhouses
Source: Glob Chall. 2026 Sep 25;10(9):e70163. doi: 10.1002/gch2.70163 (PMC13613089; doi:10.1002/gch2.70163)
Supplement: Supplementary file 1 [file GCH2-10-e70163-s001.docx]

------------- Supporting Information -------------

Influence of micro-scale surface texturing on light distribution from luminescent downshifting layers for next-generation greenhouses

Juvet N. Fru ^1^ Boris Breiner ^2^, Monica S. Saavedra ^2^, Stephanie Sheppard ^2^,
Bryce S. Richards ^1,3,*^

^1^	J.N. Fru, B. S. Richards

Institute of Microstructure Technology, Karlsruhe Institute of Technology (KIT)

Hermann-von-Helmholtz-Platz 1, 76344 Eggenstein-Leopoldshafen, Germany

^2^	Boris Breiner, Monica S. Saavedra, Stephanie Sheppard
Lambda Energy Ltd., Crop Technology Southern Innovation Hub, College Road, Cranfield, MK43 0AL, United Kingdom

^3^ B. S. Richards

Light Technology Institute, Karlsruhe Institute of Technology (KIT), Engesserstrasse 13, 76131, Karlsruhe, Germany

^*^	Corresponding author – Email: [bryce.richards@kit.edu](mailto:bryce.richards@kit.edu)

**S1.	Generation of mean free path (*MFP*) data**

To generate the mean free path (*MFP*) data, the normalised absorbance profile was first multiplied by the loading factor (*LF*) to obtain the loading-dependent absorbance (*A*). *MFP* was then determined using the formula [1]:

$MFP=$ $\frac{t}{ALn(10)}$	(S1)

where *t* = LDS layer thickness and *A* = −log_10_(*T*).

**S2.	Performance evaluation methods**

The figures of merit (FoM) for small-scale Luminescent Downshifting (LDS) layer performance, including downward extraction (*DE*) efficiency (%) for converted photons and unconverted photon outcoupling (*UPO*) efficiency (%), are defined in **Equations S1 and S2,** respectively. Similarly, **Equation S3** defines the red enhancement fraction (*REF*) (%) at the greenhouse floor, whereas the uniformity at the greenhouse floor was evaluated using the method described by Matsumori *et al.* [2] and illustrated in **Figure S1.**

Downward extracted (*DE*) efficiency $=\frac{Number of DE converted photons}{Number of absorbed photons *PLQY} (S2)$

The factor 1/PLQY in the definition of *DE* efficiency converts absorbed photons into emitted photons.

*UPO* efficiency = $\frac{average transmitted irradiance}{incident reference irradiance}*100\%, (S3)$

where the average downward irradiance represents the average irradiance of unconverted photons detected by a receiver positioned one micron below the bottom surface. The incident reference irradiance represents the average irradiance of unconverted photons detected by a receiver positioned one micron above the top surface of a planar, dye-free layer with the same dimensions.

$$Red enhancement fraction \left( REF \right)\left( \% \right)= \frac{N_{red, LDS}-N_{red, std}}{N_{red, std}}\times100\% (S4)$$

where $N_{red, LDS}$ represents the number of red photon counts detected at the large-scale LDS greenhouse floor, and $N_{red, Std}$ represents the number of direct red photon counts detected at the large-scale standard or dye-free planar (natural red photons) greenhouse floor.

**Figure S1.** Irradiance map of top-I Do greenhouse floor showing showing grids outside the uniformity (*U*) range (black and white) and grids within the uniformity range (coloured). Total number of black coloured grids and white coloured grids (outside range) = 28. Total number of grids = 160. *U* = (160-28)/160 = 83%.

S3.	Description of LightTools simulation files

**S3.1	SolidWorks textured LDS layer (description and file name)**

LightTools simulation model of a SolidWorks CAD design of a 5 × 5 × 0.3 cm LDS layer (LR305 dye) featuring 400 µm wide dome-shaped protruding surface textures on the top surface *File name: SuppInfo_S2.1_LightTools2026_SmallScale_LDS_Model_with_Protruding_Domes.*

**S3.2	LightTools textured LDS layer (description and file name)**

LightTools simulation file of a 5 × 5 × 0.3 cm LDS layer containing LR305 dye, with protruding pyramid surface textures (aspect ratio, *AR* = 1.414) applied on the top surface. *File name: SuppInfo_S2.2_LightTools2026_SmallScale_LDS_Model_with_Protruding_Pyramids_AR1.414.*

**S3.3	LightTools textured LDS greenhouse that is drawn in SolidWorks**

SolidWorks and LightTools model of an LDS greenhouse incorporating LR305 dye with Top-I dome surface textures (*AR* = 0.2). The model includes real-world solar irradiation conditions and was developed for optical performance analysis in the published study. *File name: SuppInfo_S2.3_LightTools2026_Greenhouse_LDS_Model_with__TopI_Domes_AR0.2*

**S4.	Dye loading optimisation**

**S4.1	Correlation between loading factor and dye loading**

**Figure S2** compares the simulated and experimental photon flux ratios (defined in Section S4.2) as a function of wavelength for a 5 × 5 × 0.3 cm LDS layer fabricated with PSX 700 Clear Coat. The simulated spectra correspond to different loading factors *LFs* and are represented by continuous lines, whereas the experimental spectrum corresponds to the same LDS layer with a dye loading (*DL*) of 750 ppm (wt) in the PSX 700 Clear Coat matrix (dashed lines). These optimization plots establish the relationship between *LF* and *DL* (ppm). The relationship was determined by comparing the quotient of the photon flux ratio value at the peak wavelength (400 nm) and that at the minimum wavelength (450 nm). The photon flux ratios the experimental and simulated samples are defined by **Equation S5 and S6**, respectively. The comparison showed peak-to-minimum quotient of 2.43 for the simulation and 2.50 for the experiment at a loading factor of 5. Therefore, a loading factor of 5 corresponds to a dye loading of 750 ppm, implying a loading factor of 1 corresponds to approximately 150 ppm.

**Figure S2.** Simulated photon flux ratio as a function of wavelength for a 5 × 5 × 0.3 cm LDS layer fabricated with PSX 700 Clear Coat at different loading factors (*LFs*) (continuous lines), together with the experimental photon flux ratio for the same LDS layer containing a dye loading (DL) of 750 ppm (wt) in the PSX 700 Clear Coat matrix (dashed lines).

**S4.2	Experimental photon flux ratio**

The reference (incident) spectrum was first measured without a sample. The transmitted flux was then measured with the sample in place, and the photon flux ratio was calculated by dividing the transmitted flux (converted and unconverted) by the reference spectrum.

$$Photon flux ratio= \frac{transmitted flux through sample}{transmitted flux without sample} (5)$$

**S4.3	Simulated photon flux ratio**

The simulated photon flux ratio is the ratio of the transmitted flux (converted and unconverted) detected by one micron distance detector below the bottom surface of the layer when actual layer is present (refractive index, *n* =1.5) to the transmitted flux on the same detector when the layer is replaced by air (*n* = 1).

Photon flux$\mathrm{ratio}= \frac{transmiited flux with sample}{transmiited flux without sample} (S6)$

**S5.	Experimental Validation**

**S5.1	LDS sample fabrication**

To validate the optical simulation results, one configuration from the small-scale LDS system (pyramids (Py) with an *AR* of 1.4 was realised experimentally. A dye-free planar siloxane (PPG PSX 700) clear-coat 5 × 5 × 0.3 cm layer was fabricated following the procedure described below. The selection of PSX 700 as the matrix material ensured enhanced long-term stability and durability, including resistance to weathering and chemical exposure. First, a planar polydimethylsiloxane (PDMS – RTV 615) substrate layer was prepared, with 15 g of RTV 615-part A being weighed into a 100 mL beaker using a precision balance. Subsequently, 1.5 g of RTV part B was added, and the mixture was stirred thoroughly using a spatula. The mixture was then transferred to a vacuum oven for degassing to remove entrapped air bubbles. Degassing proceeded for approximately 15 minutes until no visible bubbles remained. Next, the degassed PDMS mixture was poured into a polymethylmethacrylate (PMMA) frame (internal dimensions: 6.2 × 6.2 × 0.3 cm), which was used to confine the material and define the desired layer thickness during curing. The frame had been secured with tape onto the polished side of a planar silicon substrate. The untextured polished side ensured formation of a planar layer. The assembly, consisting of the PDMS and PMMA frame, was heated at 110 °C for 15 minutes and then allowed to cool naturally to room temperature. Prior to PDMS casting, the silicon substrate was coated with a perfluorooctyltriethoxysilane (PFOTS) anti-adhesion layer. This coating was applied by placing the wafer in a desiccator containing approximately 50 drops of PFOTS in a small open container (e.g. corked vial) and maintaining vacuum conditions for 1 hour. The dye-free PSX 700 layer (5 × 5 × 0.3 cm) was subsequently formed on the PDMS substrate. A total of 10 g of PSX 700 clear coat part A was weighed into a 50 mL beaker, followed by the addition of 2 g of part B. The mixture was stirred thoroughly and then degassed in a vacuum oven for 10 min until bubble-free. The degassed mixture was poured into a laser-cut PMMA frame (5 × 5 × 0.3 cm). The frame was firmly secured onto the PDMS substrate and sealed with tape to prevent leakage. The system was allowed to set overnight, followed by curing at 40 °C for six days. To fabricate textured samples, the planar silicon wafer was replaced with a textured silicon substrate, as reported by Huang *et al.* [3]. For LR305-dyed samples, 0.012 g of dye was weighed into a 50 mL beaker, and a few drops of xylene were added to dissolve the dye prior to introducing PSX 700 parts A and B into the same beaker.

**S5.2	Optical characterisation**

Optical transmittance and reflectance spectra were measured using a UV–vis–NIR spectrophotometer (Agilent Cary 7000, U.S.). The emission spectra of the LDS layers were measured using a spectrofluorometer (Edinburgh Instruments FS5) equipped with a built-in xenon lamp as the excitation source. A custom-built optical setup incorporating an integrating sphere (Labsphere Inc.), as previously reported [4], was used to measure the transmitted photon flux. A solar simulator (Sunbrick DIR-BASE-UV, G2V Optics Inc., Canada) containing an array of multi-channel LEDs provided sample illumination. The transmitted photon flux inside the integrating sphere was collected using a spectrometer (AvaSpec-ULS2048 × 64TEC-EVO, Avantes BV) via an optical fiber (BFY200MS02, Thorlabs Inc.). A LabVIEW routine semi-automated data acquisition and spectral integration. The experimental photon flux, defined in **Equation S5,** was calculated from the acquired data. Similarly, the simulated photon flux ratio was computed using the simulated transmitted flux, as defined in **Equation S6.** Validation was performed by comparing the simulated photon flux ratios with the experimental photon flux ratios.

**S5.3	Validation of results**

As discussed in Section 3.1.2, the unconverted photon outcoupling (*UPO*) efficiency of the bottom-indented (bottom-I) Py (*AR* = 1.4) is significantly lower than that of the top-indented (top-I) Py (*AR* = 1.4). To experimentally validate the simulations, the study defined the photon flux ratio consistently for both the measurements and simulations (**Equations S5 and S6**). **Figure S3a** confirms the predicted trend, showing an average difference in photon flux ratio (within the red spectral region) of 26% in the simulations and 24% in the experiments.

In contrast, dye-free and dyed planar layers exhibit identical photon flux ratios when measured from opposite directions (**Figures S4a and S4b**). This result agrees with the expected reversibility of light propagation in planar symmetric structures. Although the experimental trends agree well with the simulations, the experimental curves lie slightly below the simulated results. This discrepancy likely arises from matrix absorption that the simulations do not fully capture through the applied extinction coefficient, as well as from incomplete collection of emitted photons by the integrating sphere.

The study further validated the directional dependence of light transmission in bottom-I Py (*AR* = 1.4) and top-I Py (*AR* = 1.4) structures using UV–vis transmittance measurements performed from opposite directions for both dye-free and dyed samples (**Figure S3b**). The results show an approximately 56% reduction in transmittance for bottom-I Py (*AR* = 1.4) relative to top-I Py (*AR* = 1.4). Similarly, the simulations show a 53% reduction in *UPO* efficiency for bottom-I Py (*AR* = 1.4) relative to top-I Py (*AR* = 1.4), as shown in **Figure 5b.** The experimentally measured reduction in transmittance (56%) agrees closely with the simulated reduction in *UPO* efficiency (53%) when comparing bottom-I Py (*AR* = 1.4) and top-I Py (*AR* = 1.4).

**Figure S3.** (a) Photon flux ratio as a function of wavelength for simulated and experimental 5 × 5 × 0.3 cm luminescent downshifting layers with bottom-protruding (bottom-P) pyramid (Py; aspect ratio (*AR*) = 1.4) and top-indented (top-I) pyramid (Py; *AR* = 1.4) micro-structures, fabricated using a PSX-700A Clear Coat matrix containing Lumogen F Red 305 (LR305) dye at 750 parts per million (ppm). (b) Transmittance as a function of wavelength for dye-free and LR305-containing bottom-I Py (*AR* = 1.4) and top-I Py (*AR* = 1.4) structures measured using an ultraviolet–visible–infrared (UV–vis–IR) spectrophotometer.

**S5.4	Experimental transmission characteristics of planar LDS layers**

**Figure S4a, b** presents the experimental photon flux ratios of dye-free and dyed planar LDS layers measured under broadband illumination. The figure compares the optical transmission behaviour of planar samples fabricated with LR305 dye in a PSX-700A Clear Coat matrix and evaluates the directional dependence of light propagation through the layers. The photon flux ratio was calculated using **Equation S5** for SolSim measurements and by dividing the transmittance by 100% for UV–vis measurements (transmittance through air). Measurements obtained from opposite illumination directions overlap closely, demonstrating the reversible optical transmission behaviour of the planar LDS structure.

**Figure S4.** Experimental photon flux ratios of (a) dye-free and (b) dyed planar 5 × 5 × 0.3 cm luminescent downshifting (LDS) layers fabricated with Lumogen F Red 305 (LR305) dye in a PSX-700A Clear Coat matrix. SolSim and UV–vis denotes the solar simulator and ultraviolet–visible, respectively.

**S6.	Planar luminescent downshifting reference samples**

Planar LDS layers are first examined as a performance benchmark before presenting the results for textured LDS layers. For the LR305-doped LDS layer, 26% of emitted photons (14% *UE* and 12% *DE*) fall within the 42° critical angle and exit the layer through the escape cones (**Figure S5a**). This result agrees with the theoretical escape-cone prediction for a planar layer with a refractive index of *n* = 1.5. The remaining 74% of emitted photons undergo TIR at the top and bottom interfaces and are waveguided toward the edges. The extraction of these waveguided photons at the edge depends on the edge morphology (smooth or rough). For smooth-edged layers, approximately 48% of the waveguided photons escape through the edges, while the remaining 26% remain trapped within the LDS layer due to persistent TIR at the smooth interfaces. In contrast, edge roughening increases *EE* efficiency from 48% to 74% (**Figure S5b**) by disrupting the TIR symmetry and facilitating photon escape.

For GloGro-doped planar LDS layers with smooth edges, *DE*, *UE*, and *EE* are 12%, 13%, and 51%, respectively, yielding a total extraction of 76%. After edge roughening, these values change to 11%, 13%, and 75%, respectively, resulting in a total extraction of 99%. The total extraction efficiencies of roughened layers containing LR305 and GloGro are comparable, although the PLQY of LR305 is approximately 100% and that of GloGro is approximately 70%. This similarity arises because the extraction efficiency definition (**Equation S2**) references the number of emitted photons rather than the number of absorbed photons. This definition emphasises the transport and extraction behaviour of the layer rather than the intrinsic emission properties of the dye and is therefore independent of the PLQY.

In the LR305 system, overlap between the absorption and emission spectra leads to reabsorption events; however, reabsorbed photons are re-emitted because the PLQY is approximately 100%. In contrast, GloGro exhibits a large Stokes shift, which minimises spectral overlap and prevents reabsorption losses during photon transport despite its lower PLQY (~70%).

**Figure S5. (a)** Downward spectral irradiance of a 5 × 5 × 0.3 cm planar luminescent downshifting (LDS) layer (pink curve) and a transparent planar layer (black curve) with smooth edges, showing downward extraction (*DE*) efficiency, upward extraction (*UE*) efficiency, and edge extraction (*EE*) efficiency. **(b)** Edge spectral irradiance of a 5 × 5 × 0.3 cm planar LDS layer with smooth edges (black curve) and rough edges (red curve). For symmetric LDS layers with smooth edges, some rays remain trapped within the layer due to continuous total internal reflections (TIRs), resulting in a total extraction efficiency (*DE* + *UE* + *EE*) of 74%. Because the PLQY is 100%, 26% of photons remain trapped in continuous total internal reflections. Roughening the edges breaks symmetry and disrupts TIRs, enabling extraction of previously trapped photons and increasing *EE* from 48% to 74%.

**S7.	Effect of texturing on critical angle**

To the determine the effect of texturing on the critical angle, the following separate simulation was conducted and results presented in **Figure S6a, b.** A monochromatic, collimated cylindrical source (700 nm; 0.5 mm diameter) is placed at the centre of the LDS layers and oriented downward as shown in **Figure S6c.** The 700 nm wavelength lies outside the absorption range of the LR305 dye and therefore probes unconverted photons. The angle between the light source and the vertical is increased from 0° to 70°, and the transmitted irradiance (average irradiance on a detector 1 micron below the bottom surface) of the unconverted photons is detected and recorded. Transmissions are observed at and beyond 42° for all textured layers, whereas no transmission occurs at or beyond 42° for the planar layer. The behaviour of the planar layer is consistent with a critical angle of 42° for a material with *n* = 1.5. Surface texturing there broadens the critical angle or escape cone leading the enhancement in escape cone losses and hence *DE* efficiency.

**(c)**

**Figure S6.** Transmitted irradiance of unconverted light as a function of angle of incidence for a monochromatic (700 nm), collimated, cylindrical light source positioned at the center of (a) 5 × 5 × 0.3 cm luminescent downshifting (LDS) layers, including planar and bottom-protruding (bottom-P) textured layers with dome (Do), pyramid (Py), cone (Co), cylinder (Cyl), prism (Pri), and half-cylinder (H-Cyl) micro-structures at an aspect ratio (*AR*) of 1.0; (b) top-indented (top-I) pyramid (Py) textured layers with varying *AR*s; and (c) a schematic of the simulated setup.

**S8.	Angular distribution of converted red photons from planar and top-I Do LDS layer**

**Figure S7a, b** shows the angular intensity distribution of downward extracted-converted red photons from (a) planar and (b) top-I dome (*AR* = 0.45) LDS layers with dimensions of 5 × 5 × 0.3 cm, using LR305 dye as the luminescent material and a matrix refractive index of n = 1.5. Both profiles are approximately Lambertian, consistent with the isotropic emission of the dye molecules. The profiles measured along the two mutually perpendicular axes (0–180° and 90–270°) coincide, consistent with the symmetry of both the planar and dome-textured systems. The top-I dome (*AR* = 0.45) LDS layer exhibits greater intensity because the micro-textures enhance *DE* efficiency by broadening the escape cone, as explained in **Section S6.**

**Figure S7.** Angular intensity distribution of downward extracted converted photons from (a) a planar LDS layer and (b) an LDS layer with top-indented (top-I) dome (Do) micro-structures at an *AR* of 0.45. LDS, top-I, and Do, *AR* and LR305 denote luminescent downshifting, top-indented micro-structure, dome, and aspect ratio, Lumogen F Red 305, respectively.

**S9.	Downward extraction efficiency vs. aspect ratio for textured LDS layers across all positions and orientations**

**Figures S8a-f** illustrate how the *DE* efficiency of converted (luminescent) photons varies with *AR*, surface location, and orientation for six micro-structures: Do, Py, Co, Cyl, Pri, and H-Cyl. For completeness, this section includes all positions and orientations omitted from the main manuscript for conciseness. Micro-structures extract converted light more effectively when placed on the bottom surface than on the top surface.

**Figure S8**. Downward extraction (DE) efficiency of converted photons as a function of aspect ratio, position, and orientation [indented (I) or protruding (P)] for various micro-structures: (a) domes, (b) pyramids, (c) cones, (d) cylinders, (e) prisms, and (f) half-cylinders.

**S10.	*UPO* efficiency vs. AR for textured LDS layers across all positions and orientations**

**Figures S9a-f** illustrate how the *UPO* efficiency of photons varies with *AR*, surface location, and orientation for six micro-structures: Do, Py, Co, Cyl, Pri, and H-Cyl. All simulations were conducted at normal incidence. For completeness, this section includes all positions and orientations omitted from the main manuscript. Most micro-structures show a significant decrease in *UPO* efficiency when placed on the bottom surface, except for Cyl. Closely packed Py and Pri structures exhibit complete retroreflection because they lack straight-through rays in the flat regions between the micro-structures. Any micro-structure placed on the top surface preserves *UPO* efficiency.

**Figure S9.** Unconverted photon outcoupling (UPO) efficiency as a function of aspect ratio (AR), surface position (top or bottom), and orientation [indented (I) or protruding (P)] for different micro-structure geometries: (a) dome, (b) pyramid, (c) cone, (d) cylinder, (e) prism , and (f) half-cylinder.

**S11.	Angular distribution of unconverted photons from a planar LDS layer**

**Figure S10** presents the angular intensity distribution measured for a planar 5 × 5 × 0.3 cm LDS layer containing LR305 dye with a refractive index of *n* = 1.5. The incident light propagates straight through the layer at normal incidence (*θ* = 0°). Fresnel losses at the top and bottom surfaces reduce the average irradiance to 920 W m^-2^, corresponding to a *UPO* efficiency of 92%.

**Figure S10.** Angular intensity distribution of unconverted photons from a planar 5 × 5 × 0.3 cm LDS layer containing Lumogen F Red 305 (LR305) dye with a refractive index of n = 1.5.

**S12.	Angular intensity distribution of unconverted light in micro-structured LDS layers**

**Figure S11a-f** compares the intensity distributions of unconverted light for selected micro-structured LDS geometries with different surface profiles and *AR*s. The figure demonstrates how micro-structure shape, surface orientation, and indentation or protrusion geometry influence the angular propagation and extraction behaviour of unconverted photons in LR305 dye-based LDS layers. Only structures with gaps between adjacent micro-structures exhibit straight-through rays.

For example, in Cyl (*AR* = 0.9), most rays strike both the micro-structure surfaces and the inter-structure regions at normal incidence. Consequently, the straight-through peak intensity reaches approximately 920 W m^-2^. In contrast, top-I Do (*AR* = 0.45), top-I Co (*AR* = 0.45), and top-P H-Cyl (*AR* = 0.3) exhibit lower straight-through intensities than Cyl because non-zero angles of incidence refract and scatter part of the incoming light. Nevertheless, these structures still maintain a straight-through component. Rays striking the apex regions remain normally incident, and both the dome and cone geometries contain clear gaps between adjacent structures. As a result, the dome structures produce greater straight-through intensity than the half-cylinder structures.

Top-I Py (*AR* = 0.45) and top-I Pri (*AR* = 0.45) lack inter-structure gaps and contain uniformly sloped surfaces. Consequently, these geometries do not exhibit a straight-through component and instead refract light at relatively uniform angles. The scattered light is less noticeable in top-I Do (*AR* = 0.45) and top-P H-Cyl (*AR* = 0.3) because the straight-through component dominates the intensity distribution.

**Figure S11.** Intensity distributions of unconverted light (100% direct) in 5 × 5 cm LDS layers for selected micro-structure geometries: (a) top-indented (top-I) dome (Do; *AR* = 0.45), (b) top-indented (top-I) pyramid (Py; *AR* = 0.45), (c) top-indented (top-I) cone (Co; *AR* = 0.45), (d) bottom-indented (bottom-I) cylinder (Cyl; *AR* = 0.9), (e) top-indented (top-I) prism (Pri; *AR* = 0.45), and (f) top-protruding (top-P) half-cylinder (H-Cyl; *AR* = 0.3). *AR* denotes aspect ratio; top-I and bottom-I denote indented (inward-facing) micro-structures on the top and bottom surfaces, respectively, whereas top-P denotes protruding (outward-facing) micro-.structures on the top surface.

**S13.	Angular distribution of unconverted light without straight-through rays**

**Figure S12a-f** presents the intensity distributions of unconverted light for selected micro-structured LDS geometries in which the straight-through peaks are weak or absent. The figure highlights the subtle scattering and refraction effects produced by different micro-structure shapes, orientations, and *AR*s in LDS layers. By suppressing the dominant straight-through component, these distributions reveal the angular redistribution of unconverted photons more clearly. When the dominant peak at 0° is removed for top-I Do, top-I Co, and bottom-I Cyl, the scattering patterns become more apparent. The dome structure exhibits an egg-shaped loop because its curved surface contains continuously varying slopes and surface normals. In contrast, the cone structure shows a more uniform angular deviation consistent with its constant surface slope. The cylindrical structure does not exhibit noticeable scattering, as shown in **Figure S12d.**

For top-I Py and top-I Pri, the scattering patterns become more visible after reducing the intensity-axis scale rather than removing the 0° peak. These results confirm the presence of small-angle scattering in top-I Do (*AR* = 0.45), top-I Py (*AR* = 0.45), top-I Co (*AR* = 0.45), bottom-I Cyl (*AR* = 0.9), and top-P H-Cyl (*AR* = 0.3). Planar surfaces contain a single surface normal. Do structures contain infinitely many surface normals with continuously varying orientations in all directions, which produces the continuous scattering loop observed in **Figure S12a.** Square Py contain five distinct surface normals. Although the four side faces share the same tilt angle, they point in four different directions, producing the discrete scattering directions observed in **Figure S12b.** Co contain infinitely many surface normals at a constant tilt angle that rotate around the central axis. Cyl also contain infinitely many surface normals along the curved surface at a constant tilt angle, in addition to fixed normals on the flat surfaces. Pri-like structures contain three distinct surface normals, with two faces sharing the same tilt angle but pointing in opposite directions. H-cyl contain infinitely many surface normals that vary continuously in angle along a single direction.

**Figure S12.** Intensity distributions of unconverted light in 5 × 5 cm LDS layers for selected micro-structure geometries after omitting the dominant straight-through peaks to reveal the lower-intensity redirected light: (a) top-I dome (Do; *AR* = 0.45), (b) top-I pyramid (Py; *AR* = 0.45), (c) top-I cone (Co; *AR* = 0.45), (d) bottom-I cylinder (Cyl; *AR* = 0.9), (e) top-I prism (Pri; *AR* = 0.45), and (f) top-P half-cylinder (H-Cyl; *AR* = 0.3). *AR* denotes aspect ratio; top-I and bottom-I denote indented (inward-facing) micro-structures on the top and bottom surfaces, respectively, whereas top-P denotes protruding (outward-facing) micro-structures on the top surface.

**S14.	Effect of aspect ratio on unconverted-light distribution in top-I Py luminescent downshifting layers**

**Figure S13a-f** illustrates the intensity distributions of unconverted light in top-I Py LDS layers with different *AR*s. The figure demonstrates how increasing *AR* influences the angular scattering behaviour of unconverted photons. As the *AR* increases, the surface slope becomes steeper, which increases the refraction angle at the top surface. This effect subsequently increases the internal incidence angle at the bottom surface of the LDS layer and leads to larger extraction angles for the transmitted light.

**Figure S13.** Intensity distributions of unconverted light in 5 × 5 cm LDS layers with top-indented (top-I) pyramid (Py) micro**-**structures at different aspect ratios (*AR*s): (a) *AR* = 0.3, (b) *AR* = 0.45, (c) *AR* = 0.9, (d) *AR* = 1.0, (e) *AR* = 1.4, and (f) *AR* = 1.8.

**S15.	*AR*–dependent angular intensity distribution of converted red in Top-I Py LDS layers**

**Figure S14a-f** illustrates the intensity distributions of converted light in top-I Py LDS layers with different *AR*s. The figure demonstrates how variations in *AR* influence both the magnitude and angular direction of converted-photon extraction. Changes in *AR* modify the Py slope and local surface normals, which alter the angle of incidence of isotopically emitted (internally generated) photons at the textured inner top surface. As a result, the angular distribution of the emitted light varies with *AR*.

Sharp spikes characterise the faceted structures with constant-slope surfaces; however, these spikes become less pronounced in the approximately uniform elongated loop observed at AR = 1. At *AR* = 1, the Py sidewalls form angles of approximately 45°, causing the Py structure to retroreflect most low-angle emitted rays. Consequently, the intensity near 0° increases, which reduces the sharpness of the spikes.

**Figure S14.** Intensity distributions of converted red photons in 5 × 5 cm LDS layers with top-indented (top-I) pyramid (Py) micro-structures at different aspect ratios (*AR*s): (a) *AR* = 0.3, (b) *AR* = 0.45, (c) *AR* = 0.9, (d) *AR* = 1.0, (e) *AR* = 1.4, and (f) *AR* = 1.8.

**S16A. Effect of micro-structure width on downward extraction efficiency**

**Figure S15a** shows the dependence of the *DE* efficiency of converted light on micro-structure width for the investigated surface geometries at a fixed *AR*. The micro-structure width was varied from 0 to 100 µm while maintaining the corresponding *AR* for each geometry. A width of 0 µm represents the planar, untextured reference surface.

For all investigated geometries, *DE* efficiency increases sharply with the introduction of the micro-structures and then approaches a plateau. Beyond a width of approximately 5 µm, further increases in micro-structure width up to 100 µm produce little or no meaningful change in *DE* efficiency for a given geometry and *AR*. This result demonstrates that, within the investigated geometrical-optics regime, the extraction performance becomes largely insensitive to the absolute micro-structure width once the characteristic feature size exceeds approximately 5 µm.

**Figure S15A.** Dependence of the downward extraction efficiency of converted light on micro-structure width for the investigated surface geometries at a fixed aspect ratio (*AR*). Top-I and bottom-I denote indented (inward-facing) micro-structures on the top and bottom surfaces, respectively, while top-P denotes protruding (outward-facing) micro-structures on the top surface. Do, Py, Co, Cyl, Pri, and H-Cyl denote dome, pyramid, cone, cylinder, prism, and half-cylinder micro-structures, respectively.

**S16B.	Thickness-dependent downward extraction efficiency of textured LDS layers**

**Figure S15B** shows the effect of LDS slab thickness on the *DE* efficiency and *EE* efficiency for a top-indented dome (top-I Do) micro-structure with an *AR* of 0.45 and a feature width of 10 µm. *DE* efficiency remains nearly constant as the LDS layer thickness increases to approximately 100 µm, indicating that surface micro-structures, rather than layer thickness, predominantly govern photon extraction within this range. In this relatively thin-layer regime, the increase in propagation distance remains small, allowing internally trapped photons to interact efficiently with the textured interface with minimal additional optical losses, including edge losses. Consequently, the extraction probability remains relatively insensitive to thickness. At greater thicknesses, however, the longer propagation distance between successive interactions with the textured surface reduces the probability of favourable extraction within a given optical path and increases edge losses, as indicated by the corresponding increase in edge extraction efficiency. These effects likely contribute to the observed decrease in *DE* at greater thicknesses, consistent with the findings of Xu *et al.* [5].

**Figure S15B.** Downward extraction efficiency and edge extraction efficiency as a function of luminescent downshifting (LDS) slab thickness for a top-indented (top-I) dome (Do) micro-structure with an aspect ratio (*AR*) of 0.45 in a 5 × 5 cm LDS layer containing Lumogen F Red 305 (LR305) dye and having a refractive index (*n*) of 1.5. The dome (Do) feature width is 10 µm.

**S17.	Angular distribution of unconverted light in a dye-free planar greenhouse at normal incidence (sun is directly overhead)**

**Figure S16** illustrates the angular average irradiance distribution of unconverted photons at the floor of a model LDS greenhouse with a planar roof and walls under normal solar incidence. The figure shows that most rays propagate directly through the roof, with Fresnel reflections at the top and bottom roof surfaces reducing the transmitted irradiance reaching the floor to 920 W m^-2^ (92%). The results also demonstrate that tilting the roof by 20° produces only no change in Fresnel losses, consistent with previously reported behaviour for planar and dome-textured LDS thin films [6].

**Figure S16.** Angular average irradiance distribution of unconverted photons at the floor of a model LDS greenhouse with a planar roof and walls when the sun is normal to the ground or directly overhead the greenhouse.

**S18.	Seasonal solar altitude and azimuth profiles in Karlsruhe, Germany**

**Figure S17a, b** illustrates the variation in solar altitude (elevation) and azimuth angles throughout the day on March 21, June 21, September 21, and December 21 in Karlsruhe, Germany. The figure highlights the seasonal changes in solar position and solar noon throughout the year. Larger azimuthal angles indicate that the sun is positioned closer to the southern direction.

**Figure S17.** Solar altitude (a) and azimuthal (b) as a function of time of day on March 21 (solar noon ≈ 12:24), June 21 (solar noon ≈ 13:24), September 21(solar noon ≈13:24), and December 21 (solar noon ≈ 12:24) in Karlsruhe, Germany.

**S19.	Effect of roof micro-structure geometry and *AR* on greenhouse floor irradiance when the sun is normal to the ground (sun directly overhead)**

**Table S1** summarises the average irradiance of unconverted photons reaching the greenhouse floor for different LDS roof micro-structure configurations under normal solar incidence. The table compares the effects of micro-structure geometry, surface texturing, orientation, and *AR* on floor irradiance within the model greenhouse. For each micro-structure, the investigated *AR* range corresponded to the region in which the associated LDS layers exhibited minimal reductions in *UPO* efficiency in **Figure 5.** As expected, the average floor irradiance for a standard roof without texture or dopant is 920 W m^-2^. This value is lower than 1000 W m^-2^ because of Fresnel losses at the roof surfaces. All incident light passes straight through the roof without scattering, as indicated by the intensity distribution peak of 920 W m^-2^ at 0° (**Figure S16**).

None of the optimal micro-structure configurations identified for textured LDS layers—including top-I Do (*AR* = 0.45), top-I Py (*AR* = 0.45), top-I Co (*AR* = 0.45), bottom-I Cyl (*AR* = 0.9), top-I Pri (*AR* = 0.45), and top-I H-Cyl (*AR* = 0.3)—performed optimally when applied to the model greenhouse roof. Although the transmitted irradiance of unconverted photons through these LDS layers remained close to 920 W m^-2^, the average irradiance reaching the greenhouse floor was substantially lower. This reduction occurs because scattered rays escape through the greenhouse walls after interacting with the textured roof. When the *AR* decreased to 0.2, the average floor irradiance approached 920 W m^-2^. At lower *AR* values, the scattering angles become smaller (**Figure S12**), which reduces light loss through the greenhouse walls. The selection criterion was aimed at maximising average floor irradiance.

**Table S1.** Average irradiance of unconverted photons received at the floor of the model greenhouse for different roof micro-structure geometries, textured surfaces, orientations, and aspect ratios when the sun is normal to the ground. T-Pl denotes transparent planar; top-I and bottom-I denote indented (inward-facing) micro-structures on the top and bottom surfaces, respectively, whereas top-P denotes protruding (outward-facing) micro-structures on the top surface. Do, Py, Co, Cyl, Pri, and H-Cyl denote dome, pyramid, cone, cylinder, prism, and half-cylinder micro-structures, respectively.

| **Micro-structure geometry, textured surface, and orientation** | **Aspect ratio** | **Average irradiance of unconverted photons at floor level (W m^-2^)** | **Decision** |
| --- | --- | --- | --- |
| T-Pl | 0 | 920 | Selected |
| Top-I Do | 0.20 | 885 | Selected |
|  | 0.30 | 853 | Not selected |
|  | 0.45 | 789 | Not selected |
| Top-I Py | 0.20 | 887 | Selected |
|  | 0.30 | 862 | Not selected |
|  | 0.45 | 814 | Not selected |
| Top-I Co | 0.20 | 888 | Selected |
|  | 0.30 | 866 | Not selected |
|  | 0.45 | 827 | Not selected |
| Bottom-I Cyl | 0.20 | 895 | Selected |
|  | 0.30 | 883 | Not selected |
|  | 0.90 | 801 | Not selected |
| Top-I Pri | 0.20 | 893 | Selected |
|  | 0.30 | 878 | Not selected |
|  | 0.45 | 852 | Not selected |
| Top-P H-Cyl | 0.20 | 900 | Selected |
|  | 0.30 | 884 | Not selected |

**S20.	Directional distribution of unconverted photons on the greenhouse floor when the sun is normal to the ground.**

**Figure S18a-f** presents the angular intensity distributions of unconverted photons reaching the floor of a model LDS greenhouse for different roof micro-structure geometries under normal solar incidence. Smaller propagation angles produce greater average irradiance at the greenhouse floor because fewer rays escape through the greenhouse walls. In contrast, larger propagation angles increase sidewall losses and reduce the average floor irradiance. For the symmetric micro-structures (top-I Do (*AR* = 0.2), top-I Py (*AR* = 0.2), top-I Co (*AR* = 0.2), and bottom-I Cyl (*AR* = 0.2)) the distribution curves along the north–south and east–west directions coincide. In contrast, the asymmetric structures (top-I Pri (*AR* = 0.2) and top-P H-Cyl (*AR* = 0.2)) produce noncoincident directional distributions. In particular, the top-I Pri greenhouse exhibits no extraction along the east–west direction, as indicated by the absence of the red curve in **Figure S18e.** The insets reproduce the corresponding distributions on a smaller intensity scale after omitting the dominant straight-through rays, allowing the lower-intensity scattered component to be resolved more clearly. The scattered distributions demonstrate that the roof micro-structures redirect a fraction of the unconverted photons over a broader range of propagation angles.

**Figure S18.** Intensity distribution of unconverted photons at the floor of a model luminescent downshifting (LDS) greenhouse for roofs with (a) top-I dome (Do; *AR* = 0.2), (b) top-I pyramid (Py; *AR* = 0.2), (c) top-I cone (Co; *AR* = 0.2), (d) bottom-I cylinder (Cyl; *AR* = 0.2), (e) top-I prism (Pri; *AR* = 0.2), and (f) top-P half-cylinder (H-Cyl; *AR* = 0.2) textures when the sun is normal to the ground. The insets show the corresponding intensity distributions on a smaller intensity scale after omitting the dominant straight-through rays, thereby revealing the lower-intensity scattered-light distribution more clearly. *AR* denotes aspect ratio; top-I and bottom-I denote indented (inward-facing) micro-structures on the top and bottom surfaces, respectively, whereas top-P denotes protruding (outward-facing) micro-structures on the top surface.

**S21.	Angular distribution of converted photons at the greenhouse floor with the sun normal to the ground.**

**Figure S19a-g** shows the angular intensity distributions of converted photons reaching the greenhouse floor for different LDS roof micro-structures under normal solar incidence. The distributions directly influence the spatial redistribution of converted red photons on the greenhouse floor and therefore help explain the *REF* trends discussed previously. The planar LDS greenhouse exhibits an approximately Lambertian distribution of converted photons, which produces relatively uniform but less intense distribution compared with planar LDS layer (**Figure S14a**). This is because the converted photons are emitted over a broad angular range (isotopically), thus only a fraction (smaller angular range) reaches the floor efficiently, while many photons escape through the greenhouse walls.

Among the textured configurations, top-I Do and bottom-I Cyl exhibit broader and more forward-directed angular distributions of converted photons. These distributions redirect a larger fraction of emitted photons toward the greenhouse floor while limiting photon escape through the sidewalls. Consequently, these structures produce the highest *REF* values and the strongest enhancement on the greenhouse floor. The broader angular enhancement also improves spatial redistribution of converted light across the greenhouse interior. In contrast, top-I Py, top-I Co, and top-I Pri exhibit narrow and highly directional enhancement peaks. Although these structures locally enhance converted-photon intensity, many photons propagate at larger lateral angles and escape through the greenhouse walls before reaching the floor. This behaviour explains their relatively low or even negative *REF* values despite enhanced extraction from the LDS layer itself.

The top-P H-Cyl structure exhibits intermediate behaviour. Its angular distribution is broader than those of Py-, Co-, and Pri-based structures but narrower than those of top-I Do and bottom-I Cyl. As a result, it produces moderate enhancement at the greenhouse floor. Overall, the figure demonstrates that efficient greenhouse performance depends not only on the extraction efficiency of converted photons from the LDS layer but also on the angular redistribution of those photons within the greenhouse geometry. Structures that direct converted photons toward the floor while minimizing sidewall escape produce the highest *REF* values and the most effective greenhouse illumination.

**Figure S19.** Angular intensity distribution of converted photons at the floor of a model luminescent downshifting (LDS) greenhouse for (a) planar, (b) top-I dome (Do; *AR* = 0.2), (c) top-I pyramid (Py; *AR* = 0.2), (d) top-I cone (Co; *AR* = 0.2), (e) bottom-I cylinder (Cyl; *AR* = 0.2), (f) top-I prism (Pri; *AR* = 0.2), and (g) top-P half-cylinder (H-Cyl; *AR* = 0.2) roofs using Lumogen F Red 305 (LR305), with the sun normal to the ground. *AR* denotes aspect ratio; top-I and bottom-I denote indented (inward-facing) micro-structures on the top and bottom surfaces, respectively, and top-P denotes protruding (inward-facing) micro-structure on top surface. E–W and N–S denote the east–west and north–south directions, respectively.

**S22A.	Seasonal floor angular intensity distribution of unconverted light in LDS greenhouses at solar noon in Karlsruhe**

**Figure S20a-l** compares the intensity distributions of unconverted light on the greenhouse floor for planar, bottom-I Cyl (*AR* = 0.2) and top-I Do (*AR* = 0.2) LDS greenhouse configurations at solar noon on March 21, June 21, September 21, and December 21 in Karlsruhe, Germany. The angle at which light reaches the greenhouse floor is inversely related to the solar elevation angle. In December, when the solar elevation is approximately 17.2°, light reaches the floor at an angle of approximately 65°. In March and September, the solar elevation increases to approximately 42.4°, reducing the floor-incidence angle to approximately 50°. In June, the solar elevation reaches approximately 64.4°, and the floor-incidence angle decreases further to approximately 24°. Larger floor-incidence angles reduce the average irradiance because a greater fraction of the transmitted rays escape through the greenhouse walls before reaching the floor

**Figure S20.** Intensity distribution of unconverted light at the greenhouse floor for (a–c) planar, (d–f) bottom-I cylinder (Cyl; *AR* = 0.2), (g–i) top-I dome (Do; *AR* = 0.2), and luminescent downshifting (LDS) greenhouses on March 21, June 21, September 21, and December 21 at solar noon (**Figure S16**) in Karlsruhe, Germany. *AR* denotes aspect ratio; top-I and bottom-I denote indented (inward-facing) micro-structures on the top and bottom surfaces, respectively. The greenhouse measures 10 m along the east–west (E–W) direction (longer side) and 4 m along the north–south (N–S) direction (shorter side).

**Section 22B: Effect of Diffuse Illumination on Unconverted-Photon Distribution**

To assess the influence of diffuse illumination on light distribution within the greenhouse, an additional simulation was conducted under 100% diffuse irradiance using the geographical coordinates of Karlsruhe, Germany, on June 21. **Figure S21A** presents the angular intensity distribution of unconverted photons reaching the greenhouse floor for the top-indented dome (top-I Do; *AR* = 0.2) textured LDS configuration. This analysis enables comparison with the corresponding direct-illumination case and illustrates how diffuse incident light modifies the angular distribution of unconverted photons at the greenhouse floor.

**Figure S20B:** Intensity distribution of unconverted photons at the floor of the modelled luminescent downshifting (LDS) greenhouse with top-indented (top-I) dome (Do) micro-structures of aspect ratio (*AR*) = 0.2 under 100% diffuse illumination (104 W m^-2^) in Karlsruhe, Germany, on June 21. E–W and N–S denote the east–west and north–south greenhouse orientations, respectively.

**S23. Floor uniformity of unconverted light in textured LDS greenhouses, with the sun normal to the ground.**

**Figure S21a-g** compares the spatial intensity maps of unconverted photons at the floor of model LDS greenhouses with different roof micro-structure geometries under normal solar incidence. The figure highlights how roof texture geometry influences the spatial uniformity of transmitted light within the greenhouse. Uniformity (*U*) was calculated using the method described by **Figure S1.** The results demonstrate that structures with strong straight-through transmission maintain highly uniform floor illumination, whereas asymmetric geometries produce direction-dependent light redistribution and reduced uniformity near the greenhouse edges.

**Figure S21.** Intensity maps of unconverted photons at the floor of a model luminescent downshifting (LDS) greenhouse for roofs with (a) planar, (b) top-I dome (Do; *AR* = 0.2), (c) top-I pyramid (Py; *AR* = 0.2), (d) top-I cone (Co; *AR* = 0.2), (e) bottom-I cylinder (Cyl; *AR* = 0.2), (f) top-I prism (Pri; *AR* = 0.2), and (g) top-P half-cylinder (H-Cyl; *AR* = 0.2) textures with the sun normal to the ground. *AR* denotes aspect ratio; top-I and bottom-I denote indented (inward-facing) micro-structures on the top and bottom surfaces, respectively, whereas top-P denotes protruding (outward-facing) micro-structures on the top surface. The greenhouse measures 10 m along the east–west (E–W) direction (longer side) and 4 m along the north–south (N–S) direction (shorter side).

**S24.	Diurnal variation of greenhouse floor irradiance in planar and textured LDS greenhouses on June 21**

**Figure S22a-i** illustrates the spatial irradiance distributions on the greenhouse floor for planar and textured LDS greenhouses—top-I Do (*AR* = 0.2) and bottom-I Cyl (*AR* = 0.2)—at three times of day: early morning (7:24 a.m., 7.4 h), mid-morning (9:24 a.m., 9.4 h), and late morning (11:24 a.m., 11.4 h), under realistic summer conditions in Karlsruhe, Germany. Spatial irradiance distributions provide important information on uniformity (*U*), a key figure of merit in greenhouse technology because high uniformity promotes even plant growth. The calculation of *U* is described in **Figure S4.**

**Figures S22a, S22d, and S22g** show significantly lower average irradiance near the north-east end of the greenhouse during the early morning. At this time, the low solar elevation angle (**Figure S17**) causes part of the incident light to miss the greenhouse floor, producing irradiance values outside the *U* range and reducing overall *U*. Irradiance near the west wall exceeds the U range because most rays from the rising sun strike this wall directly. Reflections from the wall further increase the local irradiance.

The top-I Do greenhouse exhibits lower *U* than the planar and bottom-I Cyl greenhouses at this time. This reduction results from backward scattering by the Do micro-structures, which increases irradiance near the west wall. In contrast, bottom-I Cyl exhibits minimal scattering and therefore maintains floor *U* comparable to that of the planar greenhouse.

By late morning, the uniformity reaches 100% for the planar and top-I Do greenhouses but remains 91% for the bottom-I Cyl greenhouse. At this time, roof-reflected rays distribute more evenly across the greenhouse floor, increasing irradiance uniformity in the planar and dome-textured structures. The slightly lower uniformity for bottom-I Cyl results from scattering that reduces irradiance near the south wall.

**Figure S22.** Intensity maps at the floor of a model luminescent downshifting (LDS) greenhouse for (a–c) planar, (d–f) top-I dome (Do; *AR* = 0.2), and (g–i) bottom-I cylinder (Cyl; *AR* = 0.2) roofs on June 21 at three times of day: early morning (7:24 a.m.; 7.4 h), mid-morning (9:24 a.m.; 9.4 h), and late morning (11:24 a.m.; 11.4 h) in Karlsruhe, Germany. *AR* denotes aspect ratio; top-I and bottom-I denote indented (inward-facing) micro-structures on the top and bottom surfaces, respectively. The greenhouse measures 10 m along the east–west (E–W) direction (longer side) and 4 m along the north–south (N–S) direction (shorter side).

**References**

[1]	M.-H. Shin, H.-J. Kim, and Y.-J. Kim, "Optical modeling based on mean free path calculations for quantum dot phosphors applied to optoelectronic devices," *Optics express,* vol. 25, no. 4, pp. A113-A123, 2017.

[2]	K. Matsumori, M. Leigh, I. A. Howard, G. Huang, and B. S. Richards, "A next-generation greenhouse design for increasing solar gain in high-latitude regions," *Solar Energy,* vol. 286, p. 113196, 2025.

[3]	G. Huang *et al.*, "Broad-range spectral management towards next-generation net-zero energy greenhouses," *Energy & Environmental Science,* vol. 18, no. 21, pp. 9435-9445, 2025.

[4]	E. Madirov, D. Busko, I. A. Howard, B. S. Richards, and A. Turshatov, "Absolute quantum yield for understanding upconversion and downshift luminescence in PbF 2: Er 3+, Yb 3+ crystals," *Physical Chemistry Chemical Physics,* vol. 25, no. 17, pp. 11986-11997, 2023.

[5]	Z. Xu, M. Portnoi, and I. Papakonstantinou, "Micro-cone arrays enhance outcoupling efficiency in horticulture luminescent solar concentrators," *Optics Letters,* vol. 48, no. 1, pp. 183-186, 2022.

[6]	L. Shen *et al.*, "Increasing greenhouse production by spectral-shifting and unidirectional light-extracting photonics," *Nature Food,* vol. 2, no. 6, pp. 434-441, 2021.
